# Machine learning combining external validation to explore the immunopathogenesis of diabetic foot ulcer and predict therapeutic drugs

**DOI:** 10.1371/journal.pone.0328906

**Published:** 2025-08-01

**Authors:** Zhongwen Lu, Na An, Shouwei Sheng, Mao Hong, Pin Deng, Junde Wu, Shengji Zhang, Zhaojun Chen

**Affiliations:** 1 Beijing University of Chinese Medicine Third Affiliated Hospital, Beijing, Beijing, China; 2 Shandong University of Traditional Chinese Medicine, Jinan, Shandong, China; 3 The Third Affiliated Hospital of Beijing University of Traditional Chinese Medicine (Miyun District), Beijing, Beijing, China; 4 Institute of Basic Theory of Traditional Chinese Medicine, China Academy of Chinese Medical Sciences, Beijing, Beijing, China; University of Tabuk, SAUDI ARABIA

## Abstract

Diabetic foot ulcer (DFU) is a severe complication of diabetes, often leading to amputation due to poor wound healing and infection. The immune-related pathogenesis of DFU remains unclear, and therapeutic drugs are limited. This study aimed to explore the immune mechanisms of DFU and identify potential therapeutic drugs using machine learning and single-cell approaches. Through differential expression analysis of Gene Expression Omnibus (GEO) datasets, we identified 287 differentially expressed genes (DEGs), which were significantly enriched in IL-17 signaling and neutrophil chemotaxis pathways. Weighted gene co-expression network analysis (WGCNA) further pinpointed disease-associated modules containing 1,693 regulatory genes. Machine learning algorithms prioritized seven core genes—CCL20, CXCL13, FGFR2, FGFR3, PI3, PLA2G2A, and S100A8—with validation in an external dataset GSE147890 and single-cell sequencing revealing their predominant expression in neutrophils and keratinocytes. Immune infiltration analysis demonstrated significant dysregulation in DFU patients, characterized by elevated proportions of memory B cells, M0 macrophages, activated mast cells, and neutrophils. Potential therapeutic compounds were identified using the Connectivity Map database and tested through molecular docking and dynamics simulations. The study pinpointed selegiline, L-BSO, flunisolide, PP-30, and fluocinolone as promising therapeutic agents, offering new insights into the pathogenesis of diabetic foot ulcers (DFU) and potential therapeutic strategies.

## 1 Introduction

Diabetic foot ulcer (DFU) is a common and severe complication of diabetes, with a global prevalence of approximately 6.3% [1]. The lifetime risk of developing a DFU is as high as 25% [2]. Furthermore, patients with DFU face a significantly higher risk of non-traumatic lower limb amputation, which is 15–40 times greater than that of the general population [3]. The quality of life and survival rates for DFU patients after amputation are notably reduced. Previous studies [4,5] have reported a 5-year mortality rate for diabetic foot amputees ranging from 50% to 68%, which is even higher than that observed in some cancer patients. Additionally, the 10-year survival rate post-amputation is only 24% [6], highlighting the severe health risks posed by DFU to individuals worldwide. Wound healing in diabetic foot ulcers (DFU) is a complex biological process, with immunoinflammatory regulation playing a pivotal role at various stages of chronic wound healing [7]. Neutrophils are the first inflammatory cells to migrate to the wound site, where they help eliminate pathogens through multiple mechanisms and initiate both inflammatory and non-inflammatory responses [8]. Macrophages, which are crucial in the healing process, can influence wound repair depending on their polarization state (M1 or M2 macrophages) [9,10]. Additionally, chronic hyperglycemia in diabetic patients leads to oxidative stress and persistent inflammation, which weakens the immune response and increases the risk of foot infections [11]. Thus, exploring strategies to enhance the therapeutic effect of DFU by modulating the immune response could provide more effective treatments for patients.

**Fig 1:**
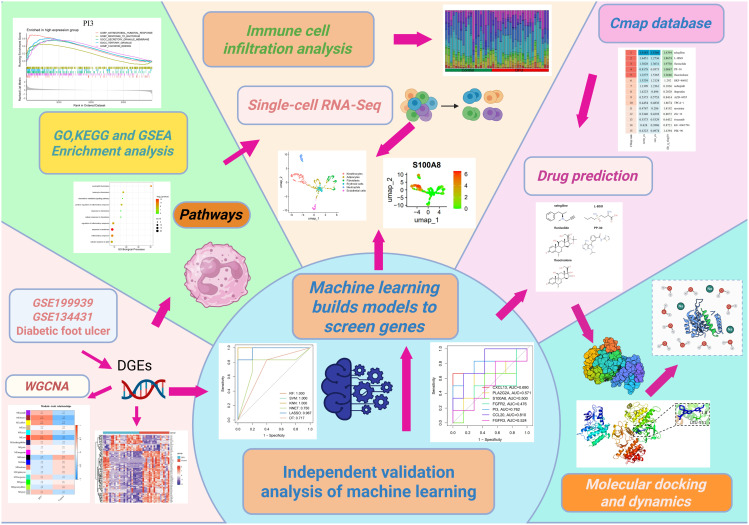
Flow chart of this study.

In the clinical management of DFU, blood glucose monitoring and control are essential components of treatment. Intensive blood glucose control has been shown to delay the onset and slow the progression of complications such as retinopathy, nephropathy, and neuropathy in insulin-dependent diabetic patients [12]. Hyperglycemia in diabetic patients induces immunosuppression by impairing both innate and adaptive immune responses. Specifically, neutrophil and macrophage dysfunction compromises pathogen clearance, while dysregulated T-cell and B-cell activity hinders adaptive immunity. These immunological disturbances collectively diminish tissue repair capacity and promote ulcer formation while impairing wound healing. Furthermore, concomitant immunosuppressive conditions—including dialysis, organ transplantation, or immunosuppressive therapy—exacerbate immune dysfunction, potentially elevating the risk of therapeutic failure in diabetic foot ulcer management [13]. Dressing materials play a key role in protecting DFU wounds by shielding them from contaminants while promoting the absorption of exudate. The effectiveness of AQAg silver dressings has been particularly demonstrated in two areas: significantly reducing ulcer depth and managing infected ulcers that require antibiotic treatment [14]. The treatment of DFU requires a multifaceted approach, including the exploration of novel compounds with potential therapeutic effects and the establishment of guidelines for drug use, thus providing more treatment options for clinical practice. Based on this knowledge, our study employed bioinformatics methods to obtain gene expression data from DFU patients and healthy controls in the GEO database. Weighted gene co-expression network analysis (WGCNA) and machine learning techniques were applied to identify immune-related markers. Additionally, through Gene Ontology (GO), Kyoto Encyclopedia of Genes and Genomes (KEGG), and Gene Set Enrichment Analysis (GSEA) enrichment analyses, key pathways associated with DFU were identified, and immune infiltration in DFU was assessed using the Cell Identification By Estimating Relative Subsets Of RNA Transcripts (CIBERSORT) algorithm. Targeted compounds were predicted using the Connectivity Map database, molecular docking and dynamic simulations were employed to further validate the results. This approach aims to provide valuable insights for the clinical treatment of DFU. The research process is illustrated in Fig 1.

## 2 Method

### 2.1 Data downloading and processing

Three original datasets (GSE134431, GSE199939, and GSE147890) were obtained from the Gene Expression Omnibus (GEO) database. GSE134431, utilizing the GPL18573 platform, contained 13 DFU patients and 8 skin tissues of diabetic patients; GSE199939, using the GPL24676 platform, included 10 Diabetic Foot Skin and 11 Non-Diabetic Foot Skin; GSE147890, based on the GPL571 platform, comprised 6 models of diabetic foot ulcer transplanted from human skin to mice and 7 models of normal human skin transplanted to mice.We annotated the probes in each dataset and converted them to standard gene names using the corresponding platform annotation files. We merged the expression profiles of the GSE134431 and GSE199939 datasets into a single training set. Batch effects were then adjusted using the ‘sva’ package to prepare for subsequent WGCNA and machine learning analyses. After standardization and normalization of the datasets, differential expression analysis between DFU and control samples was performed using the “limma” R package. Volcano plots and heatmaps were generated using the “ggplot2” package and the Heatmap function, respectively, to visualize significantly regulated genes.

### 2.2 Pathway enrichment analysis of Gene Ontology (GO) and Kyoto Encyclopedia of Genes and Genomes (KEGG)

A total of 287 differentially expressed genes (DEGs) were submitted to the Metascape database for Gene Ontology (GO) and Kyoto Encyclopedia of Genes and Genomes (KEGG) enrichment analysis, with the species set as “Homo sapiens” and the significance threshold set at P < 0.01. Using -log10(P value) as the selection criterion, the top 10 enriched terms in biological process (BP), cellular component (CC), and molecular function (MF) of the GO analysis, as well as the top 10 enriched pathways in KEGG, were visualized using bubble charts generated by R software.

### 2.3 Acquisition of immune-related genes

The ImmPort database, one of the most authoritative human immune gene resources, was used to acquire immune-related gene sets. Immune-related gene data was downloaded from the ImmPort database (https://www.immport.org/home). After removing duplicate genes, a total of 1,793 immune-related genes (IRGs) were obtained for further analysis.

### 2.4 Weighted gene co-expression network analysis (WGCNA) and identification of core modules

WGCNA is a widely used approach for modular analysis, designed to identify complex disease biomarkers or potential drug targets, uncover highly correlated gene expression patterns, and offer new insights into the underlying mechanisms of diseases. In this study, the “WGCNA” package in R software was employed to construct the co-expression network [15]. The analysis was based on the top 10,000 most variable genes from the combined dataset, with a minimum of 100 genes required for each module. The adjacency matrix was then transformed into a Topological Overlap Matrix (TOM), and multiple gene modules were identified through hierarchical clustering. To assess the association between each module and clinical features, gene significance (GS) and module membership (MM) were calculated. These metrics were used to evaluate the correlation and relevance of individual genes within the biological modules and their relationship with clinical traits. Key modules and genes with high clinical relevance were selected for further analysis.

### 2.5 Building prediction models based on multiple machine learning methods

Machine learning is one of the most commonly used tools for processing high-dimensional complex data. The R packages “glmnet,” “caret,” “Boruta” and “XGBoost” were used to build a machine learning model, using 6 machine learning algorithms including: Random Forest [16] (RF)algorithm, support vector machine-recursive feature elimination [17] (SVM-RFE)algorithm, K-Nearest Neighbors [18] (KNN) algorithm, Neural Network [19] (NNET) algorithm, least absolute shrinkage and selection operator (LASSO) and decision tree(DT)algorithm was used to predict the feature genes,the accuracy of a model is assessed, and key genes are selected for subsequent analysis.

### 2.6 Independent validation analysis of machine learning

Seven key genes associated with DFU were identified by intersecting the top-ranked genes from three machine learning models. To visualize the distribution and variability of these genes, we generated gene expression boxplots. The discriminatory power of these genes was evaluated using receiver operating characteristic (ROC) curves for both the training and test datasets. We employed the R package “rms” (version 6.3.0) to construct a Nomogram model, utilizing data from the GSE5281 and GSE132903 training sets. The predictive performance of this Nomogram was then assessed using the GSE147890 dataset as an independent external validation cohort. To thoroughly evaluate the model’s performance, we plotted calibration curves, ROC curves, and Decision Curve Analysis (DCA) curves for both the training and test sets, thereby providing a comprehensive assessment of its predictive accuracy and potential clinical utility.

### 2.7 Single-cell sequencing analysis

Process the data with the Seurat package and perform PCA analysis. Next, apply the t-SNE algorithm and UMAP (Uniform Manifold Approximation and Projection) for clustering analysis, dimensionality reduction, and cell visualization. Finally, annotate the cell clusters using the Celldex package. Finally, extract marker genes for each cell subtype from the single-cell expression profile using the threshold options of logfc.FindAllMarkers. Select immune-related genes screened by machine learning as the unique marker genes for each cell subtype.

### 2.8 Gene set enrichment analysis

Gene Set Enrichment Analysis (GSEA) uses the ‘GSEA’ package to elucidate the biological significance of characteristic genes. To achieve a standardized enrichment score for each analysis, 1000 permutations of the gene set were performed. A false discovery rate (FDR) < 0.05 indicates significant enrichment.

### 2.9 Evaluation of abundance and expression differences of immune cell subtypes

Cibersort deconvolution algorithm was used to analyze the abundance and correlation of immune infiltrating cells. The Cibersort method mainly relies on the expression matrix of 22 immune cell subtypes, which is qualitatively and quantitatively analyzed using CiberSort R script. Based on the analysis results of Cibersort, the correlation between the feature genes and the types of immune cells was analyzed. The corrplot package was used to plot the graph with P < 0.05 as the screening criterion.

### 2.10 Cmap database predicts potential drugs

A promising drug screening tool is the Connectivity Map (CMap; https://clue.io/) database. The database can predict molecularly targeted drugs based on DEGs. Intersection genes of differential and immune-related genes were imported into a database that uses the L1000 analysis platform to explore networks of drugs/small molecule compounds, genes, and disease states. In this study, we used gene expression profiles and the CMap database to predict possible chemical agents to treat DFU [20]. The results suggest that interfering with the function of specific genes has potential therapeutic effects, as the expression patterns of the corresponding genes are diametrically opposed to the disease-specific expression patterns.

### 2.11 Molecular docking verification

Molecular docking is a computational method used to predict the binding pose and affinity between proteins and ligands. For molecular docking, the 3D structures of active ingredients were obtained from the ChemSpider database (https://www.chemspider.com/). These structures were then energy-minimized using Chem3D and stored in “.pdb” format. Core genes, including CCL20 (PDB ID: 5UR7), CXCL13 (PDB ID: 5CBA), FGFR2 (PDB ID: 3RI1), FGFR3 (PDB ID: 8UDT), PI3 (PDB ID: 6ATU), PLA2G2A (PDB ID: 3U8D), and S100A8 (PDB ID: 5HLO), were retrieved from the PDB database (http://www.rcsb.org/). Using PYMOL software, water molecules and original ligands were removed from the core target proteins, and missing residues were repaired with Chimera (version 1.16). After adding hydrogen atoms and calculating the charge with Autodock, both the receptor proteins and selected compounds were saved in PDBQT format. The binding site for molecular docking was optimized by adjusting the central coordinates and docking protein size. The docking of core receptor proteins with molecule ligands was performed using Autodock Vina. For each molecular docking, 50 conformations were generated, and the best conformation was selected based on binding energy and binding site location. The final docking results were presented as heatmaps, with lower binding energy indicating a stronger affinity between the key target and core active ingredients. The interactions between the compounds and proteins were visualized using PYMOL software. Additionally, docking results were compared with reference drugs or inhibitors to assess the reliability of the predictions.

### 2.12 Molecular dynamics simulation

Molecular dynamics (MD) simulations of the ligand-receptor complex were performed using GROMACS (version 2022.2). The protein topology file was generated using the AMBER99SB-ILDN force field, and the ligand topology file was generated with the ACPYPE script utilizing the AMBER force field. The system was placed in a triclinic box, filled with TIP3P water molecules, and periodic boundary conditions were applied. Neutralization of the system was achieved by adding NaCl counter ions. Before the MD simulation, we performed energy minimization for approximately 1000 steps and then equilibrated the system using NVT and NPT ensembles for about 100 ps each.Finally, we get the Root Mean Square Deviation (RMSD), Root Mean Square Fluctuation (RMSF),Radius of Gyration(Rg) and H-bond results of these complexes. The data is visualized using Origin software.

### 2.13 Ethical approval declarations

The Gene Expression Omnibus (GEO) database is publicly accessible, and ethical approval has been obtained for all patients included in the dataset. Users are free to download the data for research purposes and to publish related findings. Since our study utilized open-source data, no ethical concerns or conflicts of interest arise.

## 3 Results

### 3.1 Identification of DEGs in DFU

**Fig 2:**
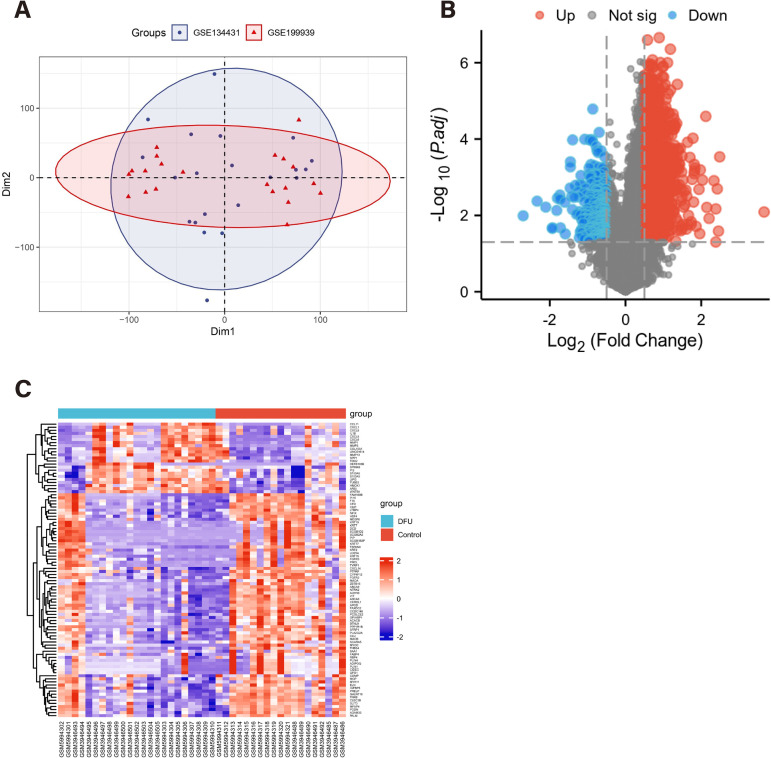
Screening differentially expressed genes (DEGs) and senescence related DEGs in diabetic foot ulcer (DFU). (A)The principal component analysis (PCA) displaying a distinct profile between GSE134431 and GSE199939. (B) The volcano plot showing upregulated (Red) and downregulated (Blue) DEGs. (C) Clustering analysis and heatmap of the DEGs between DFU and control groups.

The GSE134431 dataset was based on the GPL18573 platform, including 13 DFU samples and 8 control samples. The GSE199939 dataset is based on the GPL24676 platform, including 10 DFU samples and 11 control samples. The probes in each dataset are annotated, the expression data from the GSE5281 and GSE132903 datasets were combined into a single training set, with genes standardized according to the corresponding platform annotation files. Batch effects were then corrected using the SVA package to adjust for potential confounding variables (Fig 2A). After pretreatment and batch effect removal, a total of 287 differentially expressed genes (DEGs) were identified using the criteria of |logFC| > 1 and P < 0.05. Among these, 227 genes were up-regulated and 60 were down-regulated in DFU samples, as shown in the heatmap (Fig 2B) and volcano plot (Fig 2C).

### 3.2 GO function and KEGG pathway enrichment analysis

**Fig 3:**
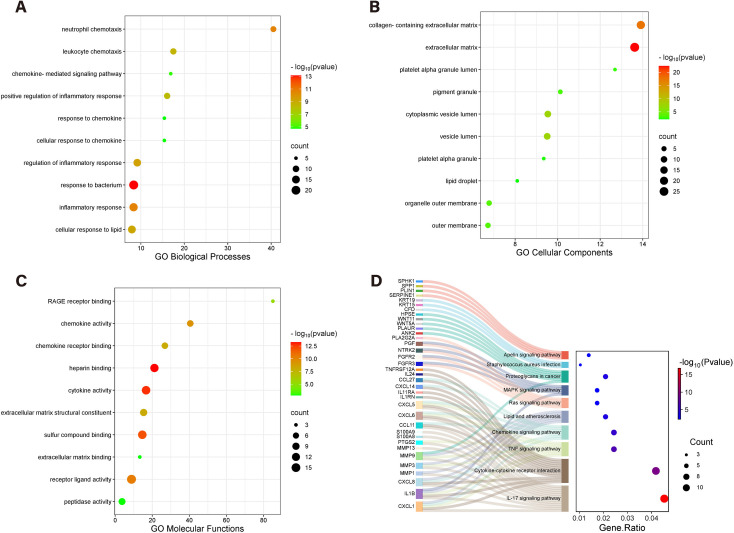
GO and KEGG Enrichment Analysis of DFU. (A-C) The bubble plots of the GO enrichment of DEGs, including biological process, cellular component, and molecular function. (D) The Sankey diagram showing the KEGG enrichment analysis of DEGs.

The analysis revealed 690 enriched GO terms and 44 KEGG pathways. The GO enrichment analysis showed that the significantly enriched biological processes mainly focused on inflammatory responses, neutrophil chemotaxis, cellular responses to lipids, and related biological processes (Fig 3A); the enriched cellular components were related to extracellular matrix, lipid droplets, and mitochondrial outer membranes, etc. (Fig 3B); the key molecular functions included cytokine activity, chemokine receptor binding, and heparin binding, etc. (Fig 3C). The KEGG pathway analysis further indicated that the main enriched pathways were the IL-17 signaling pathway, cytokine-cytokine receptor interaction, MAPK signaling pathway, etc. (Fig 3D), comprehensively revealing the multi-dimensional roles of differentially expressed genes in inflammatory regulation, cell structure composition, and signal transduction. Detailed results of GO and KEGG enrichment can be found in S1 File.

### 3.3 Construction and module identification of co-expression network

**Fig 4:**
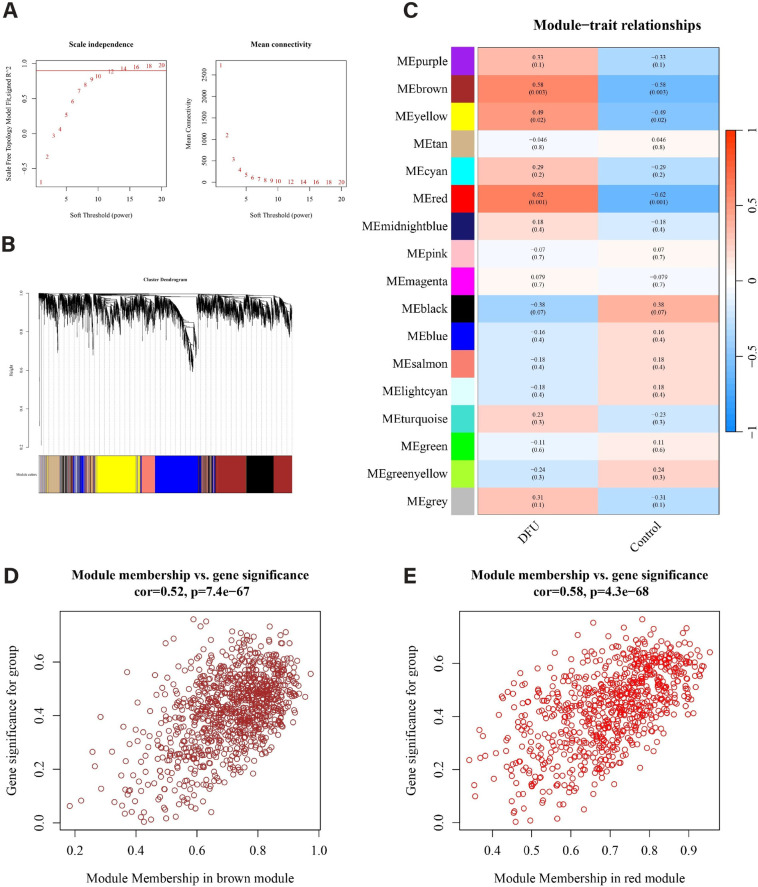
Identification of gene modules associated with DFU using WGCNA. (A) The selection of optimal soft thresholding power. (B) Gene dendrogram and modules. Gene modules associated with DFU were shown in different colors under the gene dendrogram. (C) The correlation heatmap representing the relationship between different gene modules and status of DFU. (D-E) Scatter plots showing the correlation between module membership (MM) and gene significance (GS) in the red and brown modules. WGCNA, weighted gene co-expression network analysis.

As shown in Fig 3A, the optimal soft threshold is 12. At this point, the R² value in Fig 4A (left) is approximately 0.9. Using this soft threshold, the gene modules related to DFU were identified through the hybrid dynamic shear tree algorithm (Fig 4B). The top 10,000 genes with the highest degree of variation were clustered, and a total of 17 co-expression modules were formed (Fig 4C). The Pearson correlation analysis showed that all modules were significantly correlated with clinical characteristics. Among them, the brown module (correlation coefficient 0.58, P < 0.01) and the red module (correlation coefficient 0.62, P < 0.01) had stronger correlations (Fig 4C). Significance analysis and module membership analysis indicated that the genes in the brown module (correlation coefficient 0.52, P < 0.01) and the red module (correlation coefficient 0.58, P < 0.01) had high correlations with their respective modules. Therefore, the brown and red modules were determined as key modules, and 1693 genes were selected for further research (Fig 4D and 4E).

### 3.4 Construction of the models

**Fig 5:**
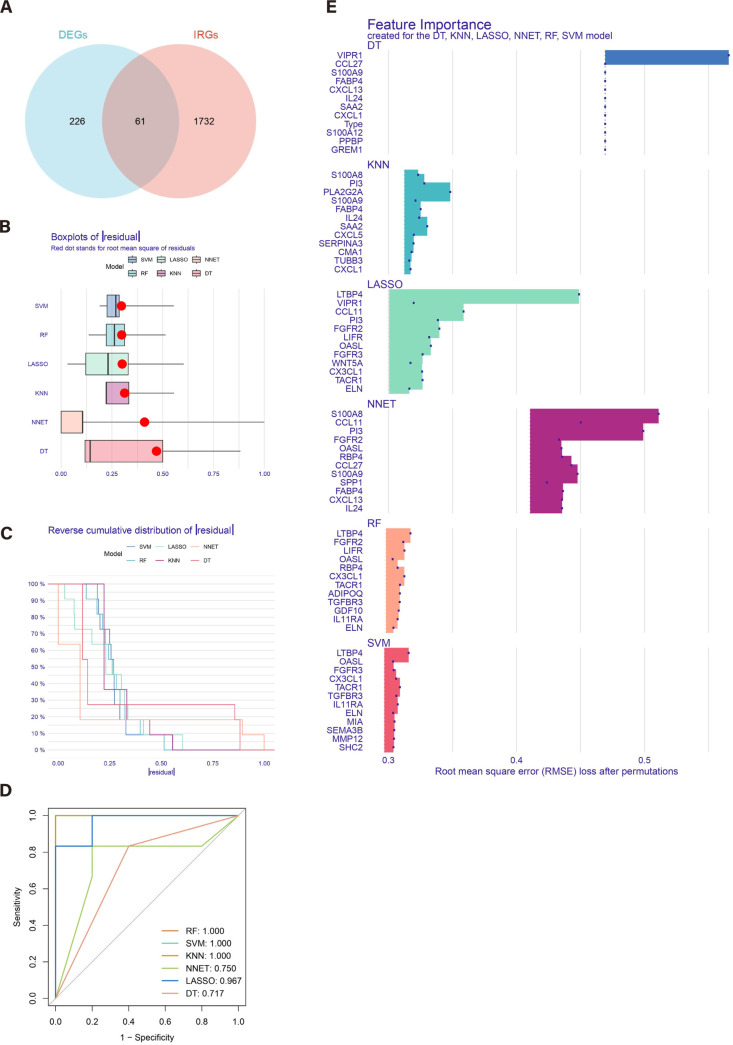
Construction of RF, SVM, KNN, NNET, LASSO and DT machine models. (A) Intersection of the differentially expressed genes and immune-related genes. (B) The cumulative residual distribution of the six models. (C) Residual Boxplots of the six machine learning models, where the red dots indicate the root mean square of the residuals. (D) ROC analysis of six machine learning models with fivefold cross-validation in the test set. (E) The important features in RF, SVM, KNN, NNET, LASSO and DT.

A total of 61 genes were identified through the intersection of the differentially expressed genes and immune-related genes (Fig 5A). Six machine learning algorithms were employed to model these 61 genes: Random Forest (RF), Support Vector Machine-Recursive Feature Elimination (SVM-RFE), K-Nearest Neighbors (KNN), Neural Network (NNET), Least Absolute Shrinkage and Selection Operator (LASSO), and Decision Tree (DT). The residual distribution results for the six models are presented in Fig 5B. Fig 5C ranks the top 10 significant feature variables for each model based on the root mean square error. The ROC curves reveal that the AUC values for the RF, SVM, and KNN models are all 1.000(Fig 5D), indicating they achieved perfect classification performance. The most important features identified by these models are shown in Fig 5E. Based on these results, we selected the RF, SVM, and KNN models for further analysis.

### 3.5 Validation analysis of machine learning

**Fig 6:**
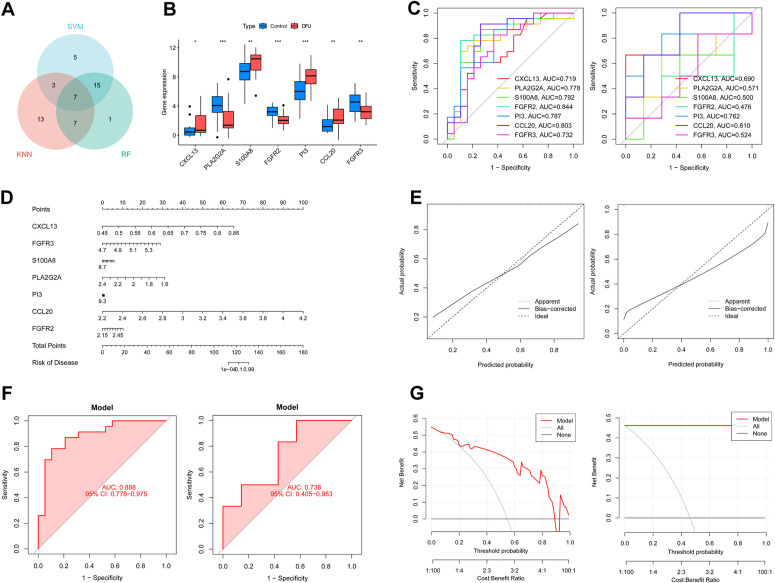
Validation analysis of machine learning of seven feature genes. (A) Intersection of the three machine learning outcomes. (B) Gene expression boxplots for 7 feature genes. (C) ROC curve of 7 feature genes (The left is the training set and the right is the test set). (D) The diagnostic nomogram based on 7 feature genes. (E) Calibration curve to evaluate the accuracy of the nomogram(The left is the training set and the right is the test set). (F)ROC of the validation Gene Expression Omnibus (GEO) data set(The left is the training set and the right is the test set).(G) Decision curve of feature genes nomogram (The left is the training set and the right is the test set).

The top 30 feature importance genes from three machine learning models were intersected, resulting in the identification of the 7 most critical genes (Fig 6A). Gene expression boxplots for these genes were generated using the R package “ggpubr” (Fig 6B). Next, we obtained the individual rating scales of these 7 feature genes in the training set and test set, and quantitatively evaluated their performance using the ROC curve, as shown in Fig 6C (the left is the training set and the right is the test set). Subsequently, we used the R package “rms” to construct the Nomogram model based on the training set data GSE5281 and GSE132903 (Fig D). To evaluate the performance of the Nomogram model, we adopted GSE147890 as an independent external validation set. The calibration curves, ROC curves and Decision Curve Analysis (DCA) curves of the training set and the test set were plotted, as shown in Figs E, F and G. The calibration curve shows that the error between the actual clustering risk of diabetic foot ulcer and the predicted risk is relatively small, and the Nomogram prediction probability is relatively close to the ideal model (Fig E). ROC analysis indicated that it had a discriminative ability with medium to high confidence (Fig F). Finally, the DCA curve analysis indicated that using nomogram for decision-making could bring clinical benefits (Fig 6G), which reflected the significant importance of these seven characteristic genes for the diagnosis and treatment of diabetic foot ulcer.

### 3.6 Overview of hub gene expression in single cells

**Fig 7:**
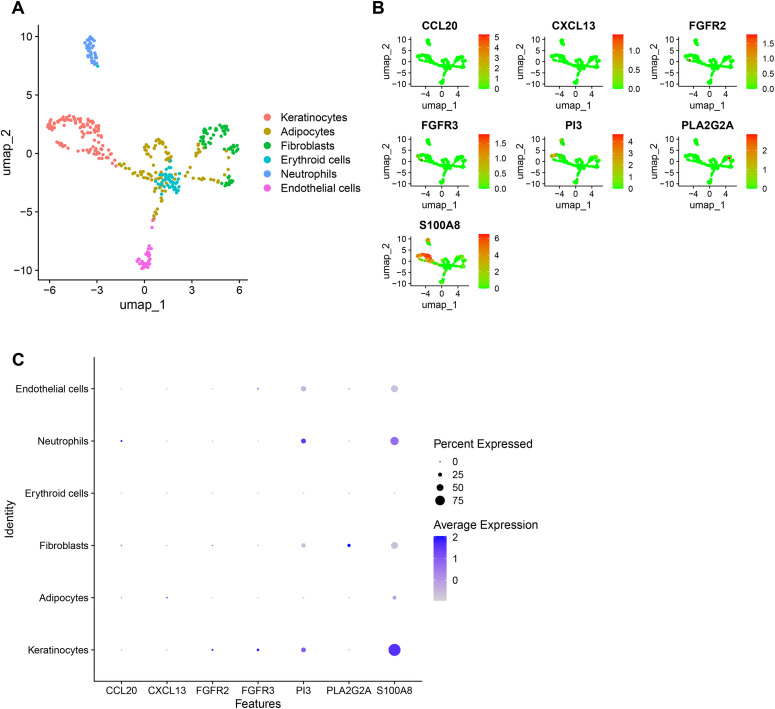
Expression profiles of hub genes in single cells. (A) Cellular subtypes of Diabetic foot ulcer. (B), (C) Scatter plots and bubble plot of the expression of the 7 hub genes.

The single-cell data of GSE248247 was downloaded and analyzed using the Seurat package. Clustering of cells was performed using the tSNE algorithm and UMAP (Uniform Manifold Approximation and Projection), and each cluster was annotated using the R package SingleR. All cells were categorized into six groups: keratinocytes, adipocytes, fibroblasts, erythroid cells, neutrophils, and endothelial cells (Fig7A). Fig7B and 7C displays the expression levels of CCL20, CXCL13, FGFR2, FGFR3, PI3, PLA2G2A, and S100A8 across the six cell types.The results showed that the key pathogenic genes of diabetic foot ulcer were mainly involved in keratinocytes and neutrophils.

### 3.7 Single-gene GSEA of characteristic genes

**Fig 8:**
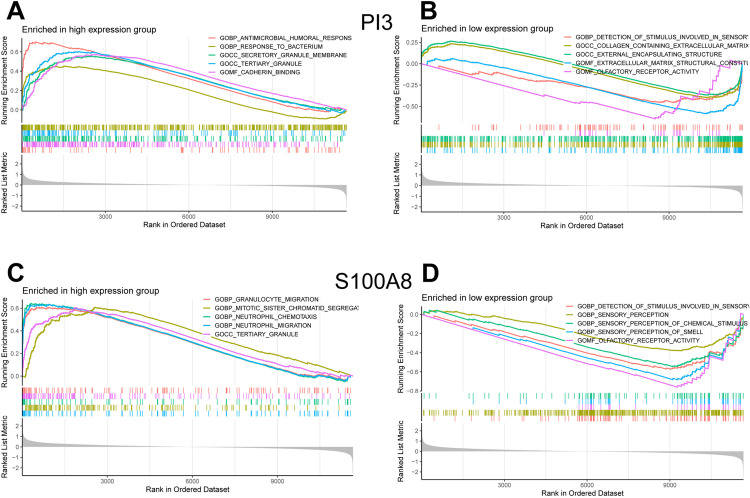
Single gene GSEA of characteristic genes. GO and KEGG enrichment analysis using GSEA for the gene PI3 and S100A8, including enriched in high expression group and low expression group.

The results of the Gene Set Enrichment Analysis (GSEA) revealed that these markers are involved in multiple pathways associated with the occurrence and progression of DFU. These pathways primarily include myeloid leukocyte migration, granulocyte migration, epidermal cell differentiation, intermediate filament organization, keratinocyte differentiation, extracellular matrix structural constituent, collagen catabolic process, epidermis development, neutrophil chemotaxis, skin development, specific granule membrane, secretory granule membrane, antimicrobial humoral response, detection of stimuli involved in sensory perception, collagen-containing extracellular matrix, receptor complex formation, translation factor activity, RNA binding, and tertiary granule formation. These pathways are all likely closely linked to the immune regulation mechanisms in DFU, the PI3 and S100A8 results were shown in the Fig 8 and the CCL20, CXCL13, FGFR2, FGFR3, and PLA2G2A GSEA analysis diagrams are shown in S2 File.

### 3.8 Immune infiltration analysis

**Fig 9:**
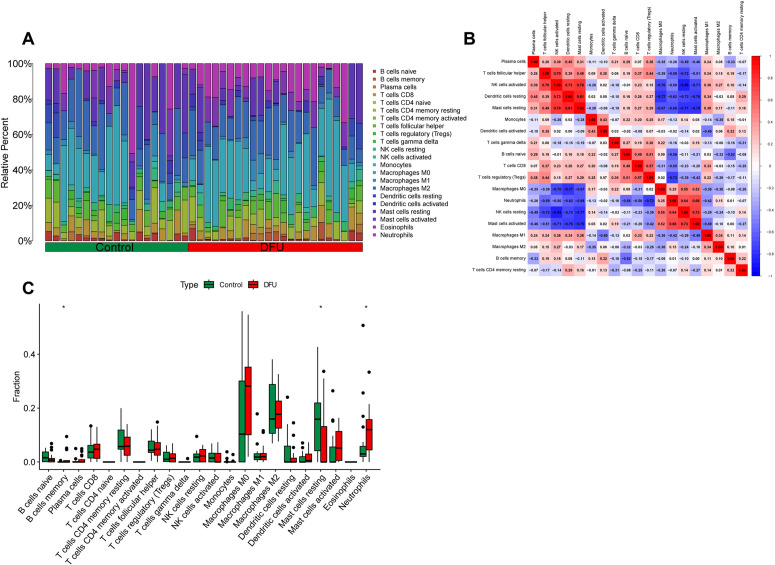
Immune cell infiltration analysis. (A) The stacked bar plot representing the different immune cell proportions in each sample. (B) The heatmap showing the correlation between different immune cells. Red represented a positive correlation, while blue represented a negative correlation. (C) The boxplot depicting the comparison of 22 types of immune cells between DCM and control groups.

The CIBERSORT algorithm was used to evaluate the immune cell abundance in DFU patients and controls. A barplot was generated to visualize the proportions of immune cells in each sample, as shown in Fig 9A. The results showed that, compared to normal tissue, DFU patient tissue had a lower proportion of naive B cells, resting mast cells, and M2 macrophages. In contrast, the proportion of memory B cells, M0 macrophages, activated mast cells, and neutrophils was higher in DFU tissue compared to the control group (Fig 9C). Additionally, correlation analysis (Fig 9B) of the 22 immune cell types revealed significant positive correlations between resting mast cells and resting dendritic cells (r = 0.81, p < 0.05), and between resting mast cells and activated mast cells (r = 0.78, p < 0.05). Conversely, negative correlations were observed between resting NK cells and activated NK cells (r = −0.88, p < 0.05), and between M0 macrophages and resting dendritic cells (r = −0.77, p < 0.05). The detailed results obtained by the CIBERSORT algorithm can be found in S3 File.

### 3.9 Screening potential drugs in the CMap database

**Fig 10:**
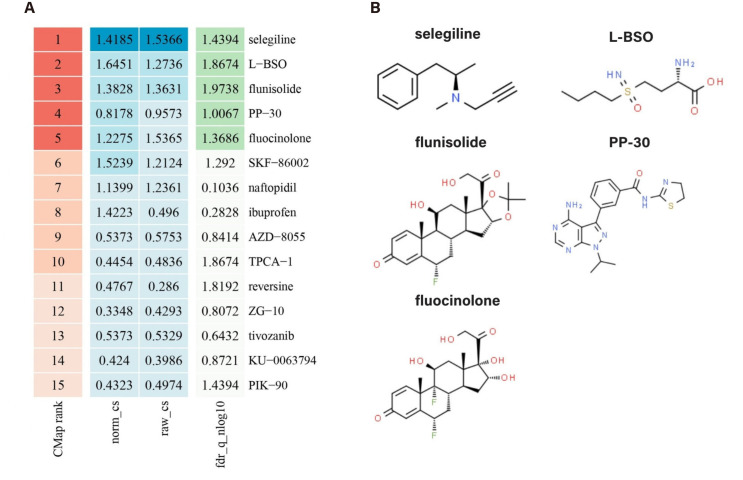
The ranking of drugs in the CMap database and the drug molecular structure. (A) Ranking and scoring of drugs in the CMap database. (B) The molecular structure of drugs.

We submitted 61 immune-related genes with differential expression to the Connectivity Map database to identify potential therapeutic compounds. The results showed that the gene expression profiles were negatively correlated with the effects of several drugs, including selegiline, L-BSO, flunisolide, PP-30, and fluocinolone. This suggests that these drugs may help alleviate or even reverse the disease state. The top 15 drugs, ranked by CMap score, are presented in Fig 9A. Additionally, the molecular structures of some of the predicted drugs are shown in Fig 10B. The detailed drug information predicted by the CMap Database can be found in S4 File.

### 3.10 Molecular docking

**Fig 11:**
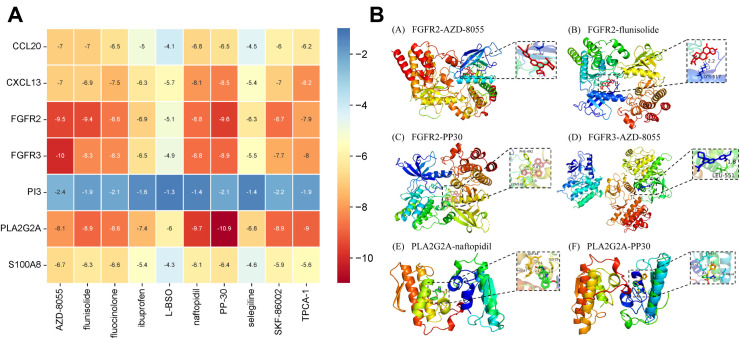
Results of molecular docking. (A) Binding energy results of molecular docking. (B) Presentation of molecular docking results.

According to receptor-ligand docking theory, docking energy is inversely proportional to binding affinity, meaning that more negative docking energies indicate stronger binding between the protein and the ligand. The results of the molecular docking analysis are shown in the Fig 11A. The six pairs of compounds and proteins with the best binding affinities are: FGFR2 and AZD-8055, FGFR2 and flunisolide, FGFR2 and PP-30, FGFR3 and AZD-8055, PLA2G2A and naftopidil, and PLA2G2A and PP-30. The docking data for these six compound-protein pairs were imported into Pymol software for visualization to analyze the interactions between the compounds and the proteins, as shown in the Fig 11B. The binding energy values for the six central therapeutic targets and their corresponding active compounds were all less than −5 kcal/mol, with at least one hydrogen bond formed between the protein and the ligand. This suggests that these compounds exhibit strong binding affinity with the key therapeutic targets and can effectively exert anti-DFU effects. Based on the above screening criteria, the key active ingredients for the treatment of DFU were identified as PP-30, AZD-8055, and flunisolide. Detailed results of molecular docking can be found in S5 File.

### 3.11 Molecular dynamics simulation

**Fig 12:**
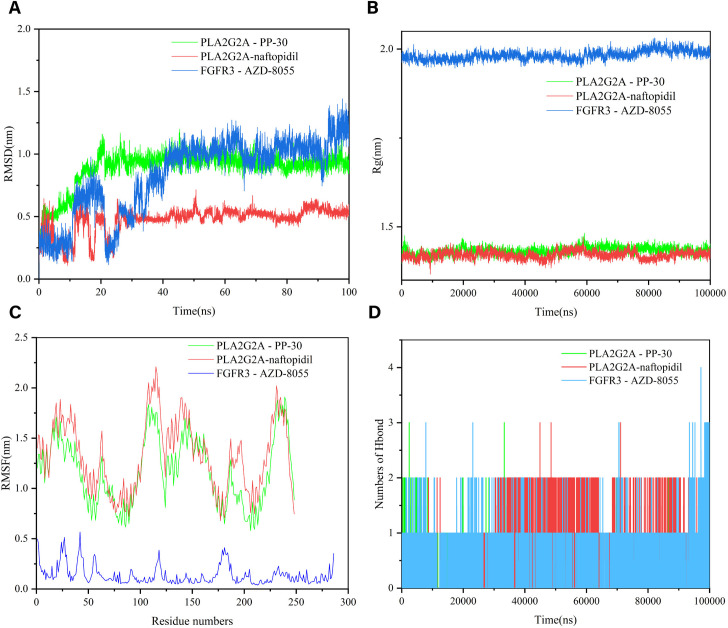
The molecular dynamics (MD) simulation of the PLA2G2A and naftopidil complex, PLA2G2A and PP-30 complex and FGFR3 and AZD-8055 complex. (A) The RMSD plot of the PLA2G2A and naftopidil complex, PLA2G2A and PP-30 complex and FGFR3 and AZD-8055 complex. (B) The Rg plot of the PLA2G2A and naftopidil complex, PLA2G2A and PP-30 complex and FGFR3 and AZD-8055 complex. (C) The RMSF plot of the PLA2G2A and naftopidil complex, PLA2G2A and PP-30 complex and FGFR3 and AZD-8055 complex. (D) The number of hydrogen bonds in the PLA2G2A and naftopidil complex, PLA2G2A and PP-30 complex and FGFR3 and AZD-8055 complex.

As shown in Fig 12A, the three combinations—PLA2G2A protein with naftopidil, PLA2G2A protein with PP-30, and FGFR3 protein with AZD-8055—demonstrate strong binding affinity and stable docking, and they were selected for molecular dynamics simulations. From the simulation results, the root mean square deviation (RMSD) of the molecules remains stable, while the RMSD of the protein-ligand complex fluctuates initially but stabilizes during the middle phase and exhibits only minor fluctuations towards the end (Fig 12A). As depicted in Fig 12B, the radius of gyration (Rg) of the complex remains stable, indicating that the overall structure of the complex is compact and robust. Additionally, root mean square fluctuation (RMSF), which reflects the residue-level flexibility of the complex, shows in Fig 12C that PLA2G2A and naftopidil, as well as PLA2G2A and PP-30, exhibit greater residue flexibility. Hydrogen bonding is a strong non-covalent interaction. The number of hydrogen bonds in the PLA2G2A and naftopidil, PLA2G2A and PP-30 complex was 0–3 in 0–100 ns (Fig 12D); and the maximum number of hydrogen bonds FGFR3 and AZD-8055 complex was 4. The hydrogen bond between the ligand and the receptor helps maintain the stability of the complex. Detailed results of dynamics can be found in S6–S9 Files.

## 4 Discussion

Diabetic foot ulcer (DFU) is a common and serious complication for individuals with diabetes, significantly impacting their quality of life and survival rates. Each year, around 18.6 million people with diabetes worldwide develop foot ulcers [21]. It is estimated that up to 34% of individuals with either type 1 or type 2 diabetes will experience foot ulcers at some point during their lifetime [22]. Moreover, about 20% of those with diabetic foot ulcers will require lower limb amputations, ranging from partial amputation (such as part of the foot) to more severe cases (such as amputations above the ankle) [23]. The financial burden of diabetic foot ulcers is substantial, highlighting the urgent need for research into more effective treatments. Current clinical approaches primarily include wound care, debridement of necrotic tissue, offloading pressure from the affected area, infection management with appropriate antibiotics, and comprehensive care [24]. However, these methods have significant limitations, frequently resulting in unsatisfactory clinical outcomes for patients.

In recent years, clinical studies have shown that the development of DFU is closely linked to immune factors, with many patients exhibiting varying degrees of immune dysfunction [25]. Tailored interventions based on the severity of the disease are crucial for improving treatment outcomes and prognosis. Some studies have indicated that the systemic immunoinflammatory index (SII) is associated with the severity of DFU in patients [26]. Thus, it is crucial to explore the pathogenesis of DFU from an immune perspective and to identify potential therapeutic agents.

Based on the GO and KEGG enrichment analysis of this study, it was found that the occurrence of DFU is related to pathways such as inflammatory response and neutrophil chemotaxis, while DFU are characterized by impaired wound healing, chronic inflammation, and a heightened risk of infection, with neutrophils playing a central role in the inflammatory phase of wound healing. The chronic hyperglycemic state typical of diabetes impairs neutrophil chemotaxis, leading to prolonged inflammation and disrupting the delicate balance required for effective wound healing. This exacerbates both inflammation and tissue damage, ultimately hindering the healing process of diabetic foot ulcers [27]. DFU is a chronic wound maintained by pathological fibroblasts and an abnormal extracellular matrix (ECM). The ECM, a complex three-dimensional network, provides both the physical scaffold for cells and the environment necessary for cell adhesion, growth, migration, and differentiation. It plays a crucial role in wound repair [28]. The activity of cytokines plays a crucial role in the development of DFU. Tumor necrosis factor-α (TNF-α) is a potent pro-inflammatory cytokine, while B cell activating factor (BAFF) is a member of the TNF-α superfamily. BAFF levels are positively correlated with TNF-α, interferon family cytokines such as IFN-α2, IL-28A/IFN-λ2, IFN-γ, and IL-10 family cytokines such as IL-19, IL-22, and IL-26. Conversely, BAFF is negatively correlated with the IL-6 receptor family, including gp130/sIL-6Rβ. Studies have shown that reducing BAFF activity can promote the healing of DFU wounds [29]. IL-17, another important pro-inflammatory cytokine, is critical in the inflammatory phase of skin healing. Research has demonstrated that IL-17 accelerates wound closure in obese mice by reducing the presence of inflammatory macrophages. This finding underscores the significant role of the IL-17 pathway in wound healing, which could offer new avenues for therapeutic intervention in DFU management [30]. In the context of bilayered living cellular constructs, the presence of fibroblasts and keratinocytes has been shown to promote epithelial stratification, enhance tensile strength, regulate the expression of cytokines and growth factors, and improve angiogenic properties. These mechanisms collectively support the healing process of DFU, ensuring more effective wound closure and tissue regeneration [31]. During the inflammatory phase of wound healing, mast cells play a crucial role by recruiting neutrophils to the injury site and secreting cytokines that activate tissue-resident macrophages. In the proliferative phase, mast cells further contribute to wound healing by promoting fibroblast proliferation through the secretion of IL-4, VEGF, and basic fibroblast growth factor (bFGF). These factors stimulate the formation of granulation tissue, facilitate cell migration, support angiogenesis, and enhance collagen maturation, ultimately accelerating the healing process of DFU [32].

Currently, no specific drugs are available to treat DFU effectively. The management of DFU typically involves blood glucose control to slow the progression of diabetes, along with surgical debridement to remove necrotic tissue. Additional strategies commonly used to prevent infection and slow DFU progression include abscess drainage, infection-preventing dressings, and bacteriological treatments [33]. A recent study describes a double-layer antibacterial collagen scaffold with enhanced structural stability and antimicrobial properties. The scaffold consists of an epidermal antibacterial collagen layer and a collagen-glycosaminoglycan matrix, specifically designed to prevent wound infections. This dressing effectively inhibits bacterial growth while promoting vascular regeneration [34]. Furthermore, studies have shown that the use of cellulose/collagen dressings can significantly increase the rate of wound healing. Due to their ability to promote angiogenesis and accelerate re-epithelialization, these dressings are particularly effective in treating refractory diabetic foot ulcers (DFUs). The benefits of cellulose/collagen dressings have been confirmed in clinical practice and trials [35].

Bioinformatics is an interdisciplinary field that integrates biology, mathematics, and information science. It involves the acquisition, processing, storage, analysis, and interpretation of biological data through the application of diverse methods and tools from multiple disciplines. By utilizing different algorithms, bioinformatics allows for the efficient analysis of large datasets and the extraction of clinically relevant information. This approach offers valuable insights into disease research and treatment [36].The application of bioinformatics methods to predict potential therapeutic drugs has opened up new treatment strategies for DFU in clinical practice and provided a new approach for the development of clinical drugs.

In this study, machine learning techniques were used to identify seven genes associated with DFU: CXCL13, PLA2G2A, S100A8, FGFR2, PI3, CCL20, and FGFR3. Among these, the expression of the CXCL13 gene was found to be elevated in DFU patients, and this gene was associated with immune response modulation [37]. S100A8 is significantly upregulated in patients with DFU [38], contributing to persistent wound inflammation and impairing the healing process [39]. Moreover, S100A8 is closely associated with the immune system, being highly expressed in the cytoplasm of neutrophils, where it plays a role in inducing neutrophil activation [40].

The results of single-cell analysis indicated that the core genes were mainly located in neutrophils and keratinocytes, Immune infiltration analysis demonstrated significant dysregulation in DFU patients, characterized by elevated proportions of memory B cells, M0 macrophages, activated mast cells, and neutrophils.

Researches show that, Keratinocytes are involved in immune responses and are very active in wound healing and angiogenesis processes. Studies have shown that impaired keratinocyte function, particularly in producing angiogenic factors, can delay the healing of diabetic foot ulcer (DFU) wounds. This finding has significant implications for DFU treatment [41].Neutrophils are a type of white blood cell that plays a significant role in the occurrence and development of DFU. In diabetes, hyperglycemia impairs neutrophil function, preventing effective participation in wound healing. This prolongs the inflammatory phase and delays recovery [42]. Studies have shown that compared with normal tissues, DFU patients have a higher proportion of M0 macrophages and activated mast cells in their tissues [43]. Moreover, the results of immune infiltration indicate that the proportion of neutrophils in the wounds of DFU patients increases. It has been shown that diabetes can induce dysfunction and disorders of neutrophils, thereby affecting the healing of DFU wounds [44].These findings indicate that immunity plays a crucial role in the pathogenesis of DFU, consistent with earlier research.

Through the screening of the Cmap database, selegiline, L − BSO, flunisolide, PP − 30, fluocinolone, Ibuprofen, naftopidil, AZD − 8055, TPCA−1 and SKF-86002 were identified as potential drugs for the treatment of DFU. Ibuprofen, one of the most commonly used nonsteroidal anti-inflammatory drugs (NSAIDs), is a non-selective inhibitor of cyclooxygenase (COX-2), an enzyme involved in prostaglandin synthesis [45]. However, systematic administration of ibuprofen can lead to adverse effects, such as reduced kidney function or gastrointestinal bleeding [46]. Clinical studies by Jorgensen et al. [47], Gottrup et al. [48], and Sibbald et al. [49] have demonstrated that dressings containing ibuprofen (Bitan-Ibu, Coloplast A/S, Humlebaek, Denmark) can significantly reduce pain without hindering wound healing. Adding ibuprofen to dressings has been shown to be particularly effective in treating infected and painful wounds [50]. Additionally, as an NSAID, ibuprofen can influence neutrophil function and lymphocyte proliferation [51]. AZD-8055 is a potent inhibitor targeting the mammalian target of rapamycin (mTOR), a critical regulator of cellular processes such as growth and metabolism. AZD-8055 has been shown to induce apoptosis in various cell lines [52]. Activation of the mTOR pathway effectively promotes both wound healing and skin regeneration [53]. Some studies have demonstrated that downregulating YAP phosphorylation and activating mTOR signaling prevents fibroblast senescence and accelerates skin wound healing in diabetic mice [54]. TPCA-1 is a novel anti-inflammatory compound that plays a significant role in modulating immune responses and inflammatory processes. By inhibiting NF-κB activation, TPCA-1 reduces the secretion of pro-inflammatory cytokines, such as tumor necrosis factor-alpha (TNF-α) and interleukin-6 (IL-6), thereby attenuating local inflammation and promoting wound healing [55,56]. Selegiline (L-deprenyl), a monoamine oxidase-B inhibitor, has been found to improve glucose homeostasis, regulate lipid metabolism, and exert anti-inflammatory effects, all of which may positively influence wound healing in patients with DFU [57]. Selegiline also reduces oxidative stress, protects the blood-brain barrier [58], and mitigates the imbalance of free radicals and antioxidants that often occurs in diabetic patients. This imbalance leads to excessive production of reactive oxygen species (ROS), which damages cells and tissues, delaying wound healing. By reducing ROS levels through its antioxidant properties, selegiline may help accelerate the healing process [59]. Additionally, selegiline has immune-modulatory effects, which can alter the phagocytic activity of blood cells, further impacting the immune response [60]. L-BSO (L-buthionine sulfoximine), an inhibitor of glutathione synthesis, increases intracellular oxidative stress, which can promote apoptosis and tissue damage [61]. In diabetic patients, heightened oxidative stress is closely associated with the development of DFU. Thus, the use of L-BSO may aid in improving the healing of diabetic foot ulcers. Interestingly, L-BSO also plays a role in regulating immune function. In the tumor microenvironment, L-BSO has been shown to enhance anti-tumor immune responses by modifying immune cell infiltration, which may contribute to improved therapeutic outcomes [62]. The development of DFU is influenced by a variety of factors, including neuropathy, vascular disease, and infection [63]. SKF-86002 is a compound that reduces inflammation by inhibiting p38α/β [64], which can help mitigate the risk of infection by modulating the inflammatory response. This mechanism offers promising new insights for the treatment of DFU.

However, there are some limitations in this study. Due to the limited number of machine learning training sets in this study and so many genes were used for modeling, an AUC value of 1 occurred. Therefore, we used external datasets for independent validation to ensure the universality of the model, to enhance the accuracy of machine learning models, additional DFU samples should be included in future research. While this paper identifies potential drugs for treating diabetic foot ulcer, clinical trials have not yet been conducted to evaluate their clinical significance. Therefore, in the future, we will conduct research on clinical efficacy and safety based on this.

## 5 Conclusion

This study provides a comprehensive investigation into the immunological mechanisms underlying diabetic foot ulcer (DFU) through bioinformatics approaches. By integrating gene expression data and immune-related gene analysis, we identified key biomarkers and pathways involved in DFU pathogenesis. The application of various machine learning algorithms enabled the selection of immune-related markers with strong potential for clinical application, as evidenced by their discriminatory power in ROC curve analysis and validation with external datasets. And the single cell analysis reveals cluster of cells. Furthermore, our exploration of immune cell infiltration and pathway enrichment offered deeper insights into the disease’s immune landscape. Notably, the study identified promising therapeutic candidates, including selegiline, PP-30, and naftopidil, through drug screening and molecular docking studies, suggesting their potential as novel treatments for DFU. These findings contribute to a better understanding of DFU biology and open new avenues for therapeutic intervention, ultimately improving patient outcomes and reducing the burden of DFU.

## Supporting information

S1 File Supplementary Material 1 GO_KEGG Enrichment. (XLSX)

S2 File Single gene GSEA of characteristic genes. (ZIP)

S3 File CIBERSORT-Results. (XLSX)

S4 File Cmap database drug. (XLSX)

S5 File Molecular docking result. (XLSX)

S6 File Dynamics result of RMSD. (XLSX)

S7 File Dynamics result of RMSF. (XLSX)

S8 File Dynamics result of Radius of Gyration. (XLSX)

S9 File Dynamics result of Hbond. (XLSX)

## References

[1] Zhang P, Lu J, Jing Y, Tang S, Zhu D, Bi Y. Global epidemiology of diabetic foot ulceration: a systematic review and meta-analysis †. Ann Med. 2017;49(2):106–16. doi: 10.1080/07853890.2016.1231932 27585063

[2] Mavrogenis AF, Megaloikonomos PD, Antoniadou T, Igoumenou VG, Panagopoulos GN, Dimopoulos L, et al. Current concepts for the evaluation and management of diabetic foot ulcers. EFORT Open Rev. 2018;3(9):513–25. doi: 10.1302/2058-5241.3.180010 30305936 PMC6174858

[3] Dutra LMA, Melo MC, Moura MC, Leme LAP, De Carvalho MR, Mascarenhas AN, et al. Prognosis of the outcome of severe diabetic foot ulcers with multidisciplinary care. J Multidiscip Healthc. 2019;12:349–59. doi: 10.2147/JMDH.S194969 31118658 PMC6506632

[4] Margolis DJ, Malay DS, Hoffstad OJ, Leonard CE, MaCurdy T, De Nava KL, et al. Incidence of diabetic foot ulcer and lower extremity amputation among medicare beneficiaries, 2006 to 2008: Data points# 2. Data points. 2011;2:4. 22049565

[5] Forde H, Wrigley S, O’Murchadha LT, Cusack L, Casserly S, Moneley D. Five-year outcomes of patients attending a diabetic foot clinic in a tertiary referral centre. Irish Journal of Medical Science (1971-). 2020;189:511–5. 10.1007/s11845-019-02108-2 31650450

[6] Soo BP, Rajbhandari S, Egun A, Ranasinghe U, Lahart IM, Pappachan JM. Survival at 10 years following lower extremity amputations in patients with diabetic foot disease. Endocrine. 2020;69(1):100–6. doi: 10.1007/s12020-020-02292-7 32281048

[7] Shi H, Yuan X, Yang X, Huang R, Fan W, Liu G. A novel diabetic foot ulcer diagnostic model: identification and analysis of genes related to glutamine metabolism and immune infiltration. BMC Genomics. 2024;25(1):125. doi: 10.1186/s12864-024-10038-2 38287255 PMC10826017

[8] Wen Q, Liu D, Wang X, Zhang Y, Fang S, Qiu X, et al. A systematic review of ozone therapy for treating chronically refractory wounds and ulcers. Int Wound J. 2022;19(4):853–70. doi: 10.1111/iwj.13687 34612569 PMC9013593

[9] Koh TJ, DiPietro LA. Inflammation and wound healing: the role of the macrophage. Expert Rev Mol Med. 2011;13:e23. doi: 10.1017/S1462399411001943 21740602 PMC3596046

[10] Wu X, He W, Mu X, Liu Y, Deng J, Liu Y, et al. Macrophage polarization in diabetic wound healing. Burns Trauma. 2022;10:tkac051. doi: 10.1093/burnst/tkac051 36601058 PMC9797953

[11] Matijevi´c T, Talapko J, Meˇstrovi´c T, Matijevi´c M, Eri´c S, Eri´c I. Understanding the multifaceted etiopathogenesis of foot complications in individuals with diabetes. World Journal Of Clinical Cases. 2023;11(8):1669. 36970006 10.12998/wjcc.v11.i8.1669 PMC10037285

[12] Control D, Group CTR. The effect of intensive treatment of diabetes on the development and progression of long-term complications in insulin-dependent diabetes mellitus. New England Journal of Medicine. 1993;329(14):977–86. 8366922 10.1056/NEJM199309303291401

[13] Uçkay I, Schöni M, Berli MC, Niggli F, Noschajew E, Lipsky BA, et al. The association of chronic, enhanced immunosuppression with outcomes of diabetic foot infections. Endocrinol Diabetes Metab. 2022;5(1):e00298. doi: 10.1002/edm2.298 34609066 PMC8754246

[14] Jude EB, Apelqvist J, Spraul M, Martini J, Silver Dressing Study Group. Prospective randomized controlled study of Hydrofiber dressing containing ionic silver or calcium alginate dressings in non-ischaemic diabetic foot ulcers. Diabet Med. 2007;24(3):280–8. doi: 10.1111/j.1464-5491.2007.02079.x 17305788

[15] Langfelder P, Horvath S. WGCNA: an R package for weighted correlation network analysis. BMC Bioinformatics. 2008;9:559. doi: 10.1186/1471-2105-9-559 19114008 PMC2631488

[16] Rigatti SJ. Random Forest. J Insur Med. 2017;47(1):31–9. doi: 10.17849/insm-47-01-31-39.1 28836909

[17] Krause L, McHardy AC, Nattkemper TW, Pühler A, Stoye J, Meyer F. GISMO--gene identification using a support vector machine for ORF classification. Nucleic Acids Res. 2007;35(2):540–9. doi: 10.1093/nar/gkl1083 17175534 PMC1802617

[18] Srisuradetchai P, Suksrikran K. Random kernel k-nearest neighbors regression. Front Big Data. 2024;7:1402384. doi: 10.3389/fdata.2024.1402384 39011467 PMC11246867

[19] Kriegeskorte N, Golan T. Neural network models and deep learning. Curr Biol. 2019;29(7):R231–6. doi: 10.1016/j.cub.2019.02.034 30939301

[20] Subramanian A, Narayan R, Corsello SM, Peck DD, Natoli TE, Lu X, et al. A Next Generation Connectivity Map: L1000 Platform and the First 1,000,000 Profiles. Cell. 2017;171(6):1437–52.e17. doi: 10.1016/j.cell.2017.10.049 29195078 PMC5990023

[21] Zhang Y, Lazzarini PA, McPhail SM, van Netten JJ, Armstrong DG, Pacella RE. Global Disability Burdens of Diabetes-Related Lower-Extremity Complications in 1990 and 2016. Diabetes Care. 2020;43(5):964–74. doi: 10.2337/dc19-1614 32139380

[22] Armstrong DG, Boulton AJM, Bus SA. Diabetic Foot Ulcers and Their Recurrence. N Engl J Med. 2017;376(24):2367–75. doi: 10.1056/NEJMra1615439 28614678

[23] McDermott K, Fang M, Boulton AJM, Selvin E, Hicks CW. Etiology, Epidemiology, and Disparities in the Burden of Diabetic Foot Ulcers. Diabetes Care. 2023;46(1):209–21. doi: 10.2337/dci22-0043 36548709 PMC9797649

[24] Armstrong DG, Tan T-W, Boulton AJM, Bus SA. Diabetic Foot Ulcers: A Review. JAMA. 2023;330(1):62–75. doi: 10.1001/jama.2023.10578 37395769 PMC10723802

[25] Zeng L, Zhang P, Fang Z, Liu D, Li H, Qu X, et al. The Construction and Analysis of Infiltrating Immune Cell and ceRNA Networks in Diabetic Foot Ulcer. Front Endocrinol (Lausanne). 2022;13:836152. doi: 10.3389/fendo.2022.836152 35909542 PMC9329527

[26] Aydın MS, Eren MA, Uyar N, Kankılıç N, Karaaslan H, Sabuncu T, et al. Relationship between systemic immune inflammation index and amputation in patients with diabetic foot ulcer. J Orthop Sci. 2024;29(4):1060–3. doi: 10.1016/j.jos.2023.07.015 37532650

[27] Nambi N, Radhakrishnan L, Prasad MK, Ramkumar KM. Neutrophil migration is a crucial factor in wound healing and the pathogenesis of diabetic foot ulcers: insights into pharmacological interventions. Current Vascular Pharmacology. 2024. 10.2174/0115701611308960241014155413 39482919

[28] Shen J, Zhao X, Zhong Y, Yang P, Gao P, Wu X, et al. Exosomal ncRNAs: The pivotal players in diabetic wound healing. Front Immunol. 2022;13:1005307. doi: 10.3389/fimmu.2022.1005307 36420273 PMC9677725

[29] Dhamodharan U, Teena R, Vimal Kumar R, Changam SS, Ramkumar KM, Rajesh K. Circulatory levels of B-cell activating factor of the TNF family in patients with diabetic foot ulcer: Association with disease progression. Wound Repair Regen. 2019;27(5):442–9. doi: 10.1111/wrr.12720 31041853

[30] Lee J, Rodero MP, Patel J, Moi D, Mazzieri R, Khosrotehrani K. Interleukin-23 regulates interleukin-17 expression in wounds, and its inhibition accelerates diabetic wound healing through the alteration of macrophage polarization. FASEB J. 2018;32(4):2086–94. doi: 10.1096/fj.201700773R 29208701

[31] Wojtowicz AM, Oliveira S, Carlson MW, Zawadzka A, Rousseau CF, Baksh D. The importance of both fibroblasts and keratinocytes in a bilayered living cellular construct used in wound healing. Wound Repair Regen. 2014;22(2):246–55. doi: 10.1111/wrr.12154 24635175 PMC4211362

[32] Tellechea A, Leal EC, Kafanas A, Auster ME, Kuchibhotla S, Ostrovsky Y, et al. Mast Cells Regulate Wound Healing in Diabetes. Diabetes. 2016;65(7):2006–19. doi: 10.2337/db15-0340 27207516 PMC4915574

[33] Jiang P, Li Q, Luo Y, Luo F, Che Q, Lu Z, et al. Current status and progress in research on dressing management for diabetic foot ulcer. Frontiers in Endocrinology. 2023;14:1221705.). 37664860 10.3389/fendo.2023.1221705 PMC10470649

[34] McGrath M, Zimkowska K, Genoud KJ, Maughan J, Gutierrez Gonzalez J, Browne S, et al. A Biomimetic, Bilayered Antimicrobial Collagen-Based Scaffold for Enhanced Healing of Complex Wound Conditions. ACS Appl Mater Interfaces. 2023;15(14):17444–58. doi: 10.1021/acsami.2c18837 37001059 PMC10103052

[35] Naomi R, Fauzi MB. Cellulose/Collagen Dressings for Diabetic Foot Ulcer: A Review. Pharmaceutics. 2020;12(9):881. doi: 10.3390/pharmaceutics12090881 32957476 PMC7558961

[36] Jin J, Guang M, Li S, Liu Y, Zhang L, Zhang B, et al. Immune-related signature of periodontitis and Alzheimer’s disease linkage. Front Genet. 2023;14:1230245. doi: 10.3389/fgene.2023.1230245 37849501 PMC10577303

[37] Fan P, Ye H, Zhu C, Xie H. Exploring the pathogenesis of osteomyelitis accompanied by diabetic foot ulcers using microarray data analysis. Medicine (Baltimore). 2023;102(43):e33962. doi: 10.1097/MD.0000000000033962 37904457 PMC10615496

[38] Shaorong Z, Xiaodong L, Qiong P, Zhaodong X, Zhuo L, Hechen H, et al. SNHG12/NFYC-AS1 Acted as the Sponge for hsa-miR-199a-5p to Promote the Expression of S100A8/S100A7/XDH and was Involved in the Progression of Diabetic Foot Ulcers. Mol Biotechnol. 2023;65(12):2038–48. doi: 10.1007/s12033-023-00692-4 36920714

[39] Singh K, Agrawal NK, Gupta SK, Sinha P, Singh K. Increased expression of TLR9 associated with pro-inflammatory S100A8 and IL-8 in diabetic wounds could lead to unresolved inflammation in type 2 diabetes mellitus (T2DM) cases with impaired wound healing. J Diabetes Complications. 2016;30(1):99–108. doi: 10.1016/j.jdiacomp.2015.10.002 26525587

[40] Sprenkeler EGG, Zandstra J, van Kleef ND, Goetschalckx I, Verstegen B, Aarts CEM, et al. S100A8/A9 Is a Marker for the Release of Neutrophil Extracellular Traps and Induces Neutrophil Activation. Cells. 2022;11(2):236. doi: 10.3390/cells11020236 35053354 PMC8773660

[41] Morgado PI, Miguel SP, Correia IJ, Aguiar-Ricardo A. Ibuprofen loaded PVA/chitosan membranes: A highly efficient strategy towards an improved skin wound healing. Carbohydr Polym. 2017;159:136–45. doi: 10.1016/j.carbpol.2016.12.029 28038742

[42] Rai V, Moellmer R, Agrawal DK. The role of CXCL8 in chronic nonhealing diabetic foot ulcers and phenotypic changes in fibroblasts: a molecular perspective. Mol Biol Rep. 2022;49(2):1565–72. doi: 10.1007/s11033-022-07144-3 35044539

[43] Jiang N, Xu C, Xu Y, Zhuo Y, Chen P, Deng S, et al. Comprehensive transcriptomic analysis of immune-related genes in diabetic foot ulcers: New insights into mechanisms and therapeutic targets. Int Immunopharmacol. 2024;139:112638. doi: 10.1016/j.intimp.2024.112638 39079197

[44] Clayton SM, Shafikhani SH, Soulika AM. Macrophage and Neutrophil Dysfunction in Diabetic Wounds. Adv Wound Care (New Rochelle). 2024;13(9):463–84. doi: 10.1089/wound.2023.0149 38695109 PMC11535468

[45] Lu Y, Liu X, Zhao J, Bie F, Liu Y, Xie J, et al. Single-cell profiling reveals transcriptomic signatures of vascular endothelial cells in non-healing diabetic foot ulcers. Front Endocrinol (Lausanne). 2023;14:1275612. doi: 10.3389/fendo.2023.1275612 38107519 PMC10722230

[46] Price P, Fogh K, Glynn C, Krasner DL, Osterbrink J, Sibbald RG. Why combine a foam dressing with ibuprofen for wound pain and moist wound healing? Int Wound J. 2007;4 Suppl 1(Suppl 1):1–3. doi: 10.1111/j.1742-481X.2007.00310.x 17394624 PMC7951788

[47] Jørgensen B, Friis GJ, Gottrup F. Pain and quality of life for patients with venous leg ulcers: proof of concept of the efficacy of Biatain-Ibu, a new pain reducing wound dressing. Wound Repair Regen. 2006;14(3):233–9. doi: 10.1111/j.1743-6109.2006.00116.x 16808800

[48] Gottrup F, Jørgensen B, Karlsmark T, Sibbald RG, Rimdeika R, Harding K, et al. Reducing wound pain in venous leg ulcers with Biatain Ibu: a randomized, controlled double-blind clinical investigation on the performance and safety. Wound Repair Regen. 2008;16(5):615–25. doi: 10.1111/j.1524-475X.2008.00412.x 19128256

[49] Sibbald RG, Coutts P, Fierheller M, Woo K. A pilot (real-life) randomised clinical evaluation of a pain-relieving foam dressing: (ibuprofen-foam versus local best practice). Int Wound J. 2007;4 Suppl 1(Suppl 1):16–23. doi: 10.1111/j.1742-481X.2007.00308.x 17394626 PMC7951211

[50] Mogrovejo-Valdivia A, Maton M, Garcia-Fernandez MJ, Tabary N, Chai F, Neut C, et al. In Vitro Microbiological and Drug Release of Silver/Ibuprofen Loaded Wound Dressing Designed for the Treatment of Chronically Infected Painful Wounds. Antibiotics (Basel). 2021;10(7):805. doi: 10.3390/antibiotics10070805 34356725 PMC8300664

[51] Chokshi R, Bennett O, Zhelay T, Kozak JA. NSAIDs Naproxen, Ibuprofen, Salicylate, and Aspirin Inhibit TRPM7 Channels by Cytosolic Acidification. Front Physiol. 2021;12:727549. doi: 10.3389/fphys.2021.727549 34733174 PMC8558630

[52] Patel RP, Thomas JR, Curt KM, Fitzsimmons CM, Batista PJ, Bates SE, et al. Dual Inhibition of Histone Deacetylases and the Mechanistic Target of Rapamycin Promotes Apoptosis in Cell Line Models of Uveal Melanoma. Invest Ophthalmol Vis Sci. 2021;62(12):16. doi: 10.1167/iovs.62.12.16 34533562 PMC8458781

[53] Chen C, Ou Q, Chen K, Liang C, Zeng X, Lin D, et al. Foam dressing and micropower vacuum dressing promote diabetic foot ulcer wound healing by activating the PI3K/AKT/mTOR pathway in rats. J Biomater Appl. 2024;39(1):40–7. doi: 10.1177/08853282241248780 38641897

[54] Wei F, Wang A, Wang Q, Han W, Rong R, Wang L, et al. Plasma endothelial cells-derived extracellular vesicles promote wound healing in diabetes through YAP and the PI3K/Akt/mTOR pathway. Aging (Albany NY). 2020;12(12):12002–18. doi: 10.18632/aging.103366 32570219 PMC7343472

[55] Bhatti FUR, Hasty KA, Cho H. Anti-inflammatory role of TPCA-1 encapsulated nanosomes in porcine chondrocytes against TNF-α stimulation. Inflammopharmacology. 2019;27(5):1011–9. doi: 10.1007/s10787-018-0542-5 30600473

[56] Wang B, Bai S, Wang J, Ren N, Xie R, Cheng G, et al. TPCA-1 negatively regulates inflammation mediated by NF-κB pathway in mouse chronic periodontitis model. Mol Oral Microbiol. 2021;36(3):192–201. doi: 10.1111/omi.12335 33768683

[57] Joung H-Y, Oh J-M, Song M-S, Kwon Y-B, Chun S. Selegiline Modulates Lipid Metabolism by Activating AMPK Pathways of Epididymal White Adipose Tissues in HFD-Fed Obese Mice. Pharmaceutics. 2023;15(11):2539. doi: 10.3390/pharmaceutics15112539 38004519 PMC10675427

[58] Pu Y, Qian F, Guo J, Sha Y, Qian Y. Selegiline Protects Against Lipopolysaccharide (LPS)-Induced Impairment of the Blood-Brain Barrier Through Regulating the NF-κB/MLCK/p-MLC Signaling Pathway. Neurotox Res. 2022;40(1):267–75. doi: 10.1007/s12640-021-00448-5 34981455

[59] Deng L, Du C, Song P, Chen T, Rui S, Armstrong DG, et al. The Role of Oxidative Stress and Antioxidants in Diabetic Wound Healing. Oxid Med Cell Longev. 2021;2021:8852759. doi: 10.1155/2021/8852759 33628388 PMC7884160

[60] Sobieszczańska A, Lis M, Suszko-Pawłowska A, Szczypka M. Clomipramine, a tricyclic antidepressant, and selegiline, a monoamine oxidase-B inhibitor, modulate the activity of phagocytic cells after oral administration in mice. J Pharm Pharmacol. 2020;72(6):836–42. doi: 10.1111/jphp.13251 32144951

[61] Martinez-Jaramillo E, Jamali F, Abdalbari FH, Abdulkarim B, Jean-Claude BJ, Telleria CM, et al. Pro-Oxidant Auranofin and Glutathione-Depleting Combination Unveils Synergistic Lethality in Glioblastoma Cells with Aberrant Epidermal Growth Factor Receptor Expression. Cancers (Basel). 2024;16(13):2319. doi: 10.3390/cancers16132319 39001381 PMC11240359

[62] Cruz A, Mota P, Ramos C, Pires RF, Mendes C, Silva JP, et al. Polyurea Dendrimer Folate-Targeted Nanodelivery of l-Buthionine sulfoximine as a Tool to Tackle Ovarian Cancer Chemoresistance. Antioxidants (Basel). 2020;9(2):133. doi: 10.3390/antiox9020133 32028640 PMC7070262

[63] Rosinha P, Saraiva M, Ferreira L, Garrido S, Carvalho A, Freitas C, et al. A Retrospective Cohort Study on Diabetic Foot Disease: Ascertainment of Ulcer Locations by Age Group. Cureus. 2022;14(8):e28189. doi: 10.7759/cureus.28189 36158367 PMC9491625

[64] Iba M, Kim C, Kwon S, Szabo M, Horan-Portelance L, Peer CJ. Inhibition of p38α mapk restores neuronal p38γ mapk and ameliorates synaptic degeneration in a mouse model of dlb/pd. Science Translational Medicine. 2023;15(695):6089. 10.1126/scitranslmed.abq6089 PMC12168722 37163617

