# Supplementary figures and images for: Machine learning combining external validation to explore the immunopathogenesis of diabetic foot ulcer and predict therapeutic drugs
Source: PLoS One. 2025 Aug 1;20(8):e0328906. doi: 10.1371/journal.pone.0328906 (PMC12316216; doi:10.1371/journal.pone.0328906)

# Enriched in high expression group

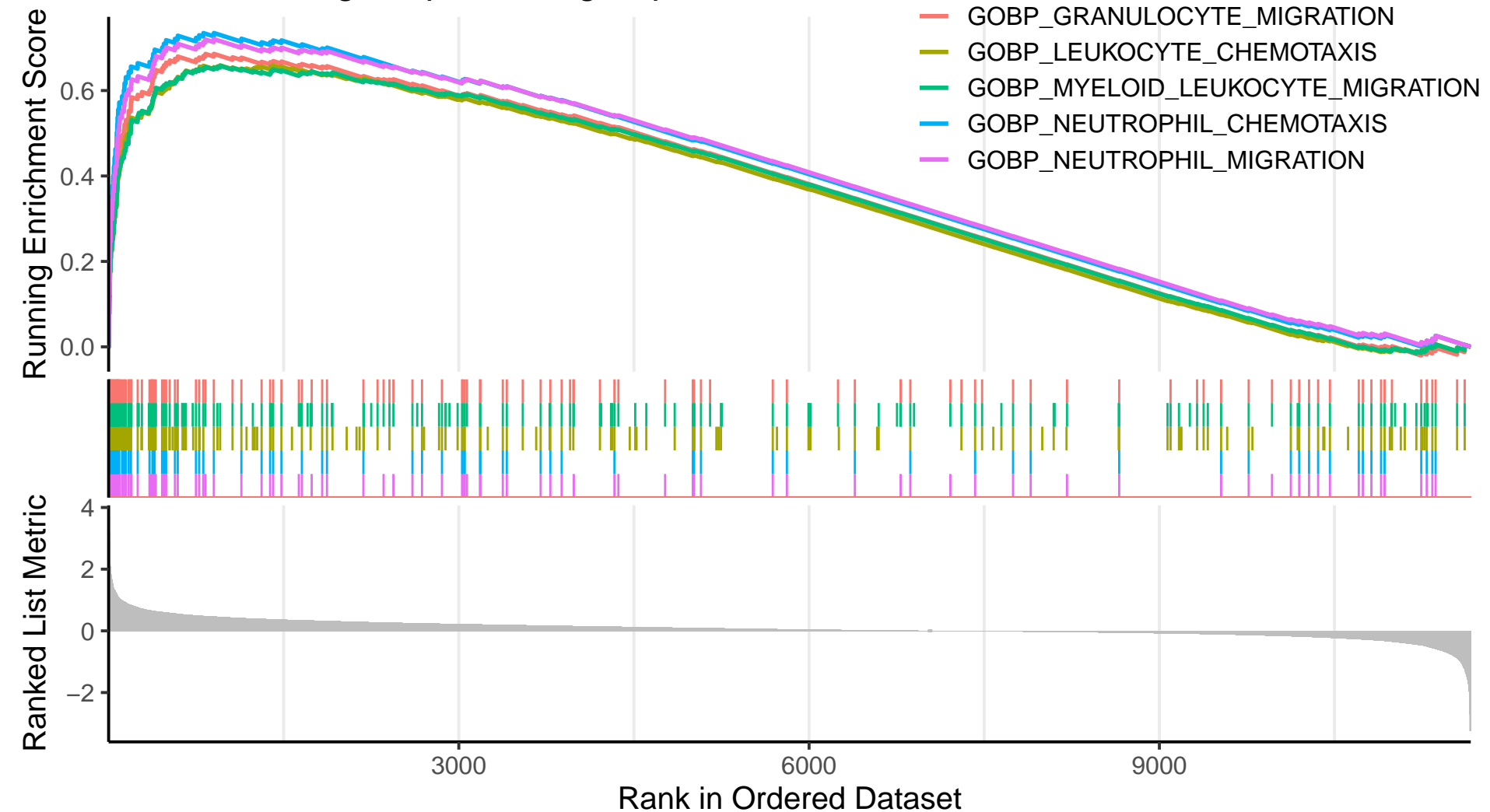

Supplement: S2 File — (ZIP) [file pone.0328906.s002.zip › Supplementary materials2/CCL20.highExp.pdf]

# Enriched in low expression group

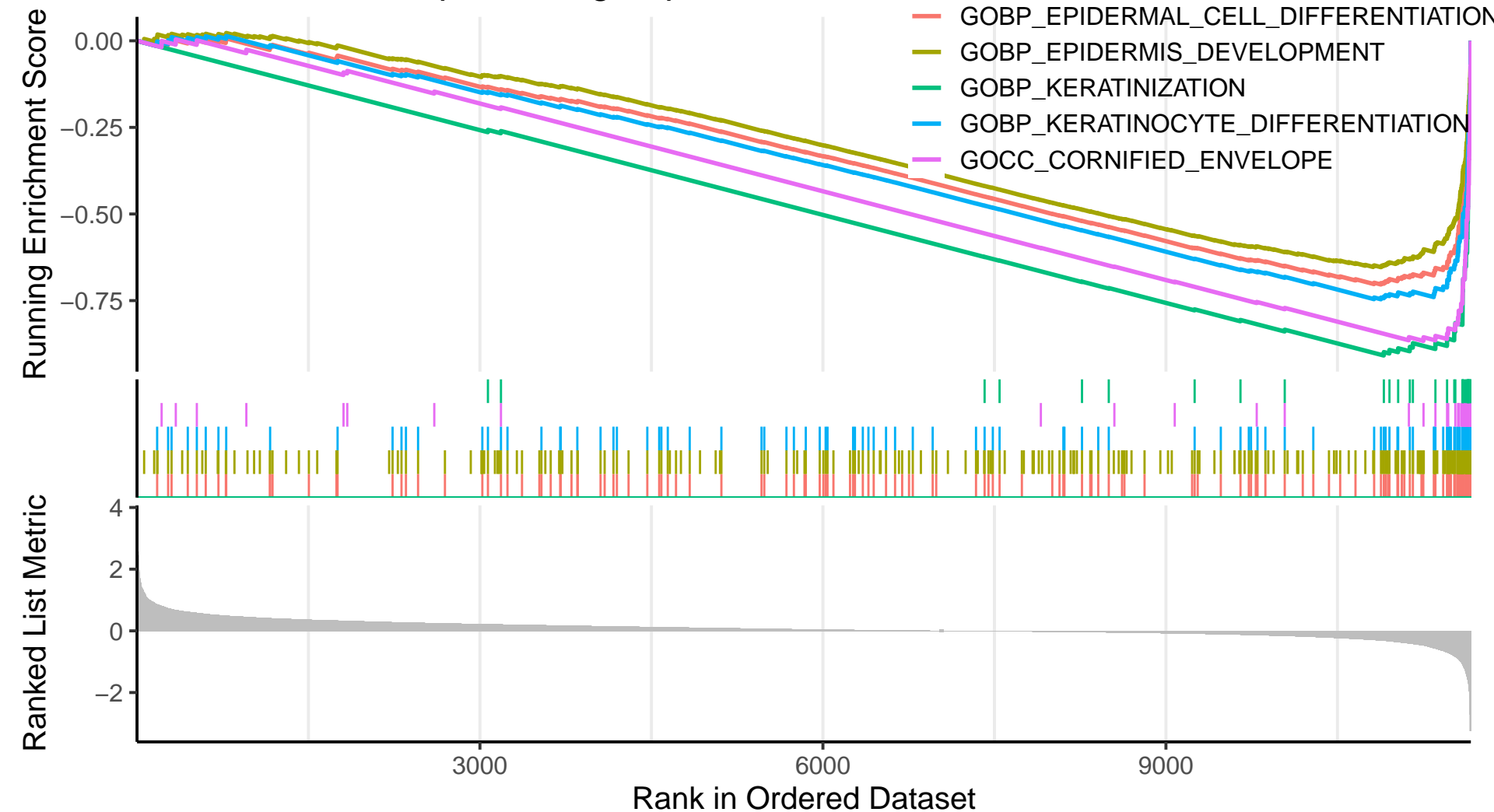

Supplement: S2 File — (ZIP) [file pone.0328906.s002.zip › Supplementary materials2/CCL20.lowExp.pdf]

# Enriched in high expression group

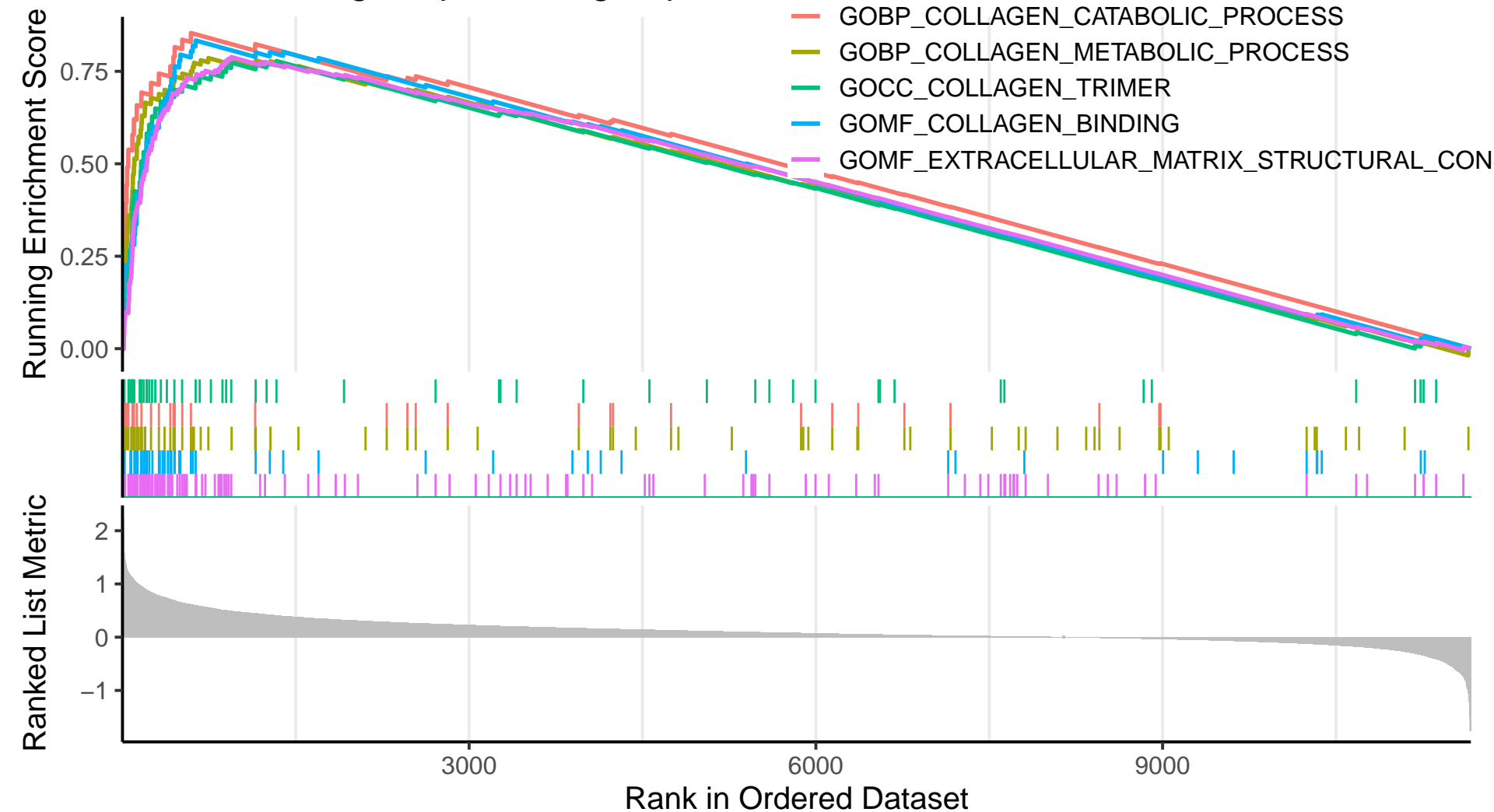

Supplement: S2 File — (ZIP) [file pone.0328906.s002.zip › Supplementary materials2/CXCL13.highExp.pdf]

# Enriched in low expression group

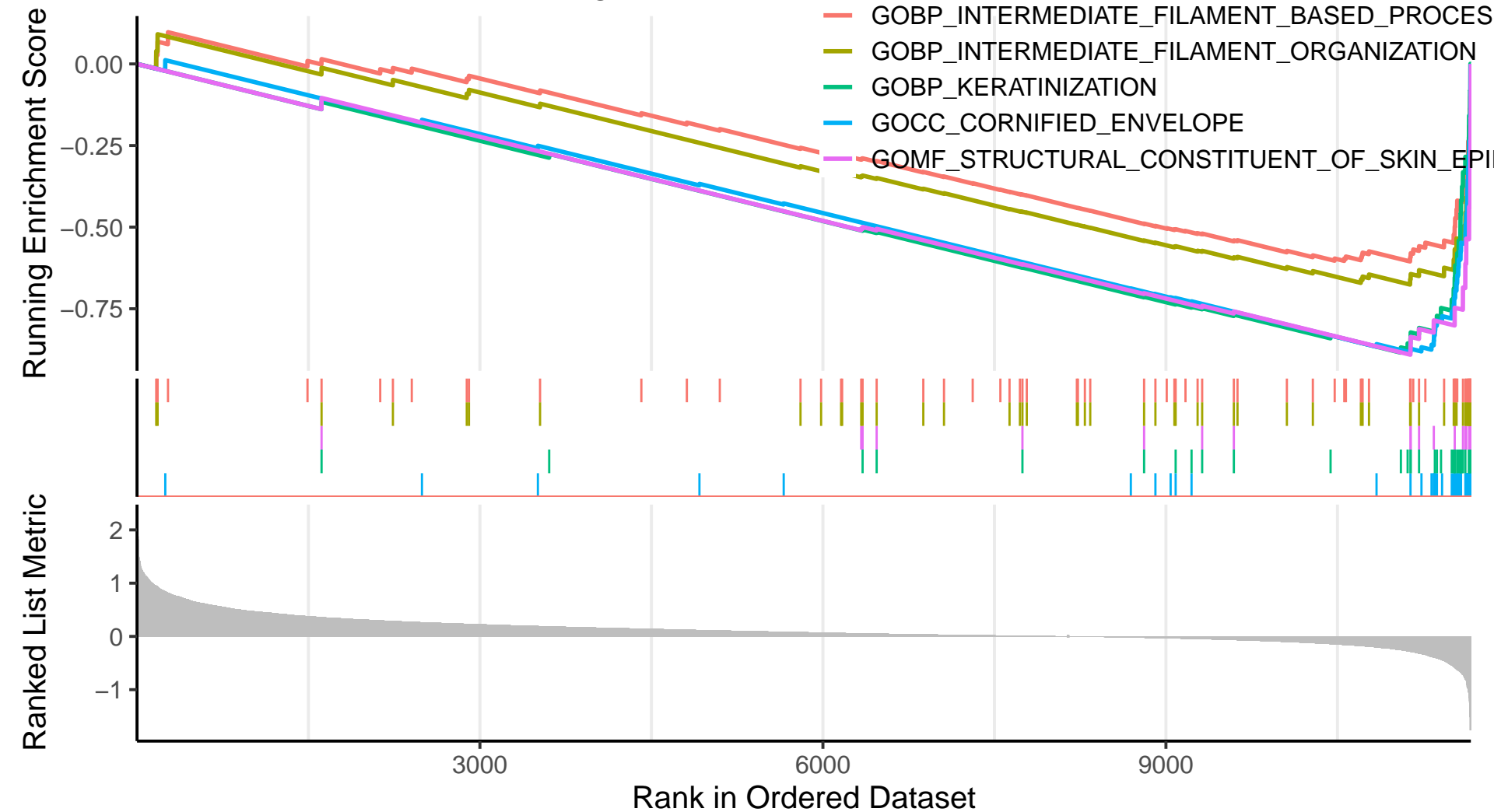

Supplement: S2 File — (ZIP) [file pone.0328906.s002.zip › Supplementary materials2/CXCL13.lowExp.pdf]

# Enriched in high expression group

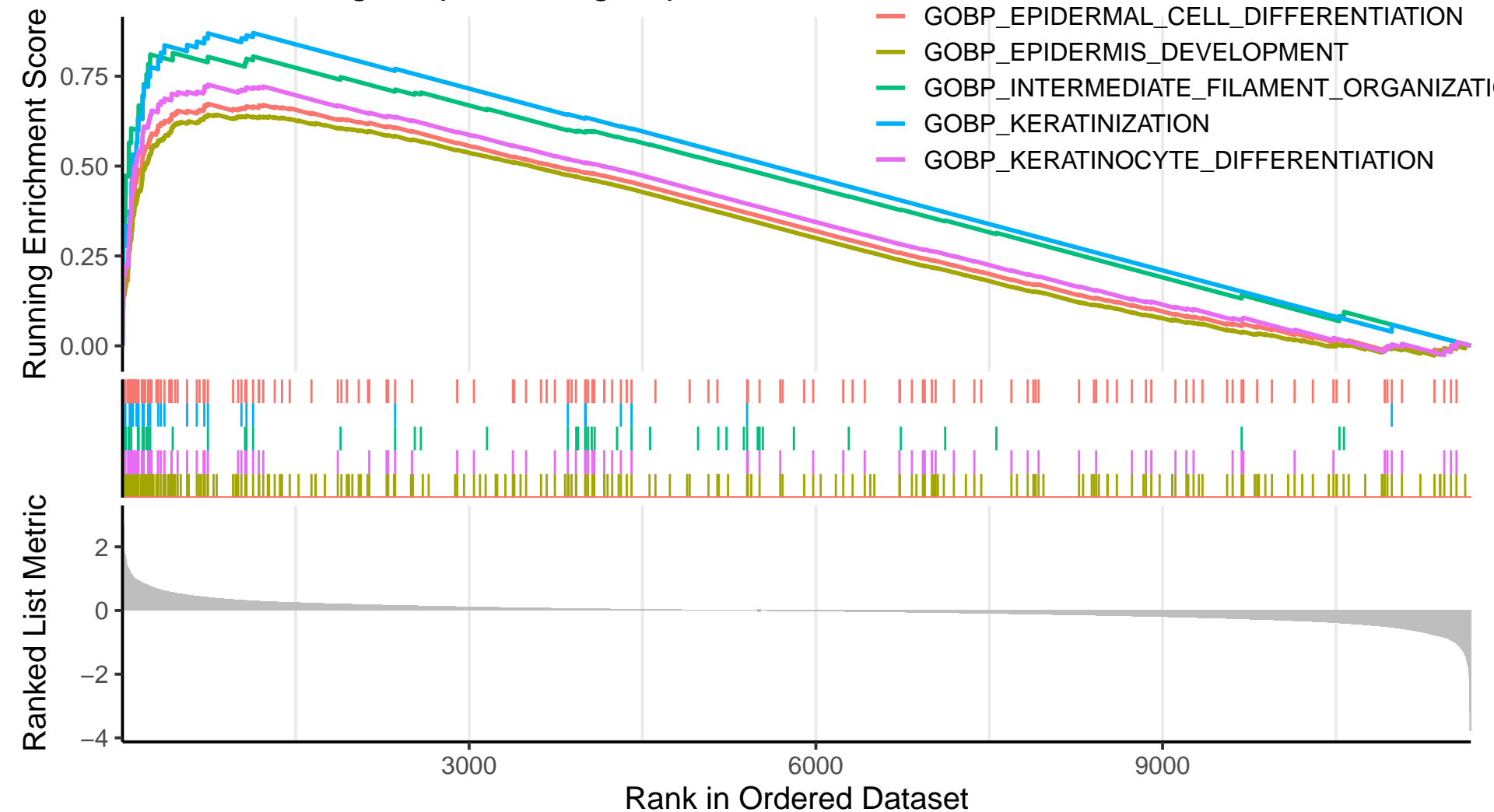

Supplement: S2 File — (ZIP) [file pone.0328906.s002.zip › Supplementary materials2/FGFR2.highExp.pdf]

# Enriched in low expression group

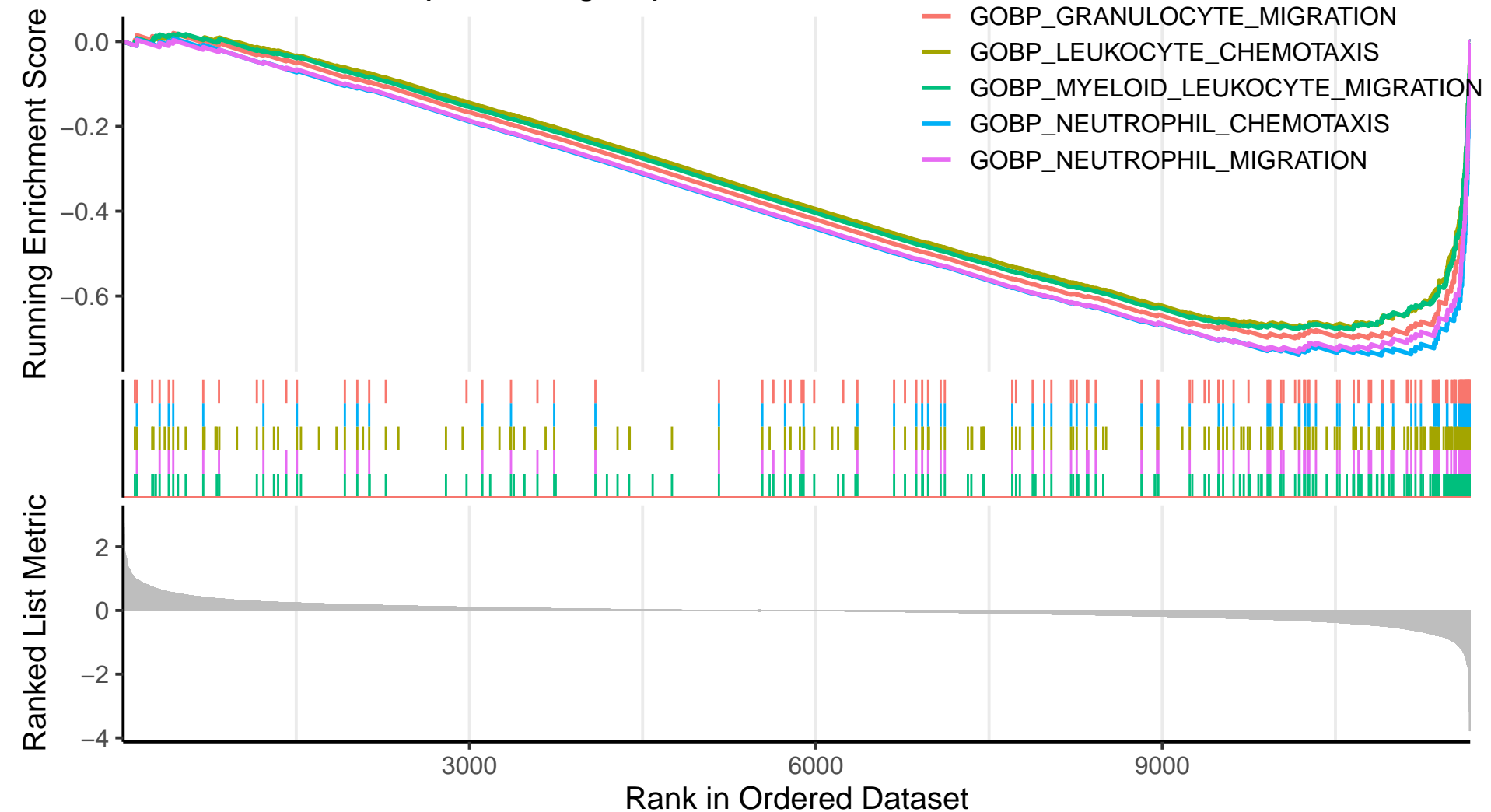

Supplement: S2 File — (ZIP) [file pone.0328906.s002.zip › Supplementary materials2/FGFR2.lowExp.pdf]

# Enriched in high expression group

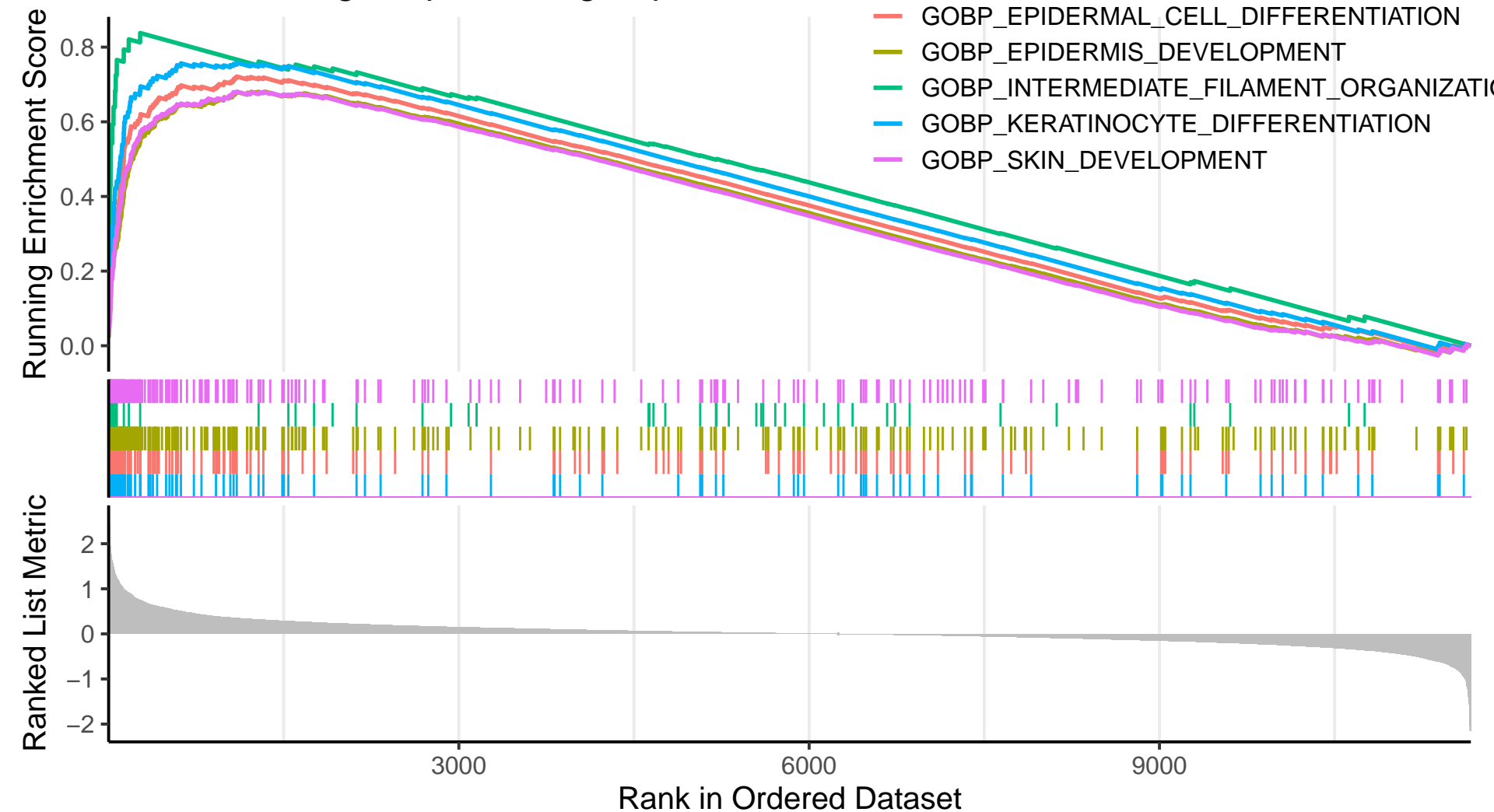

Supplement: S2 File — (ZIP) [file pone.0328906.s002.zip › Supplementary materials2/FGFR3.highExp.pdf]

# Enriched in low expression group

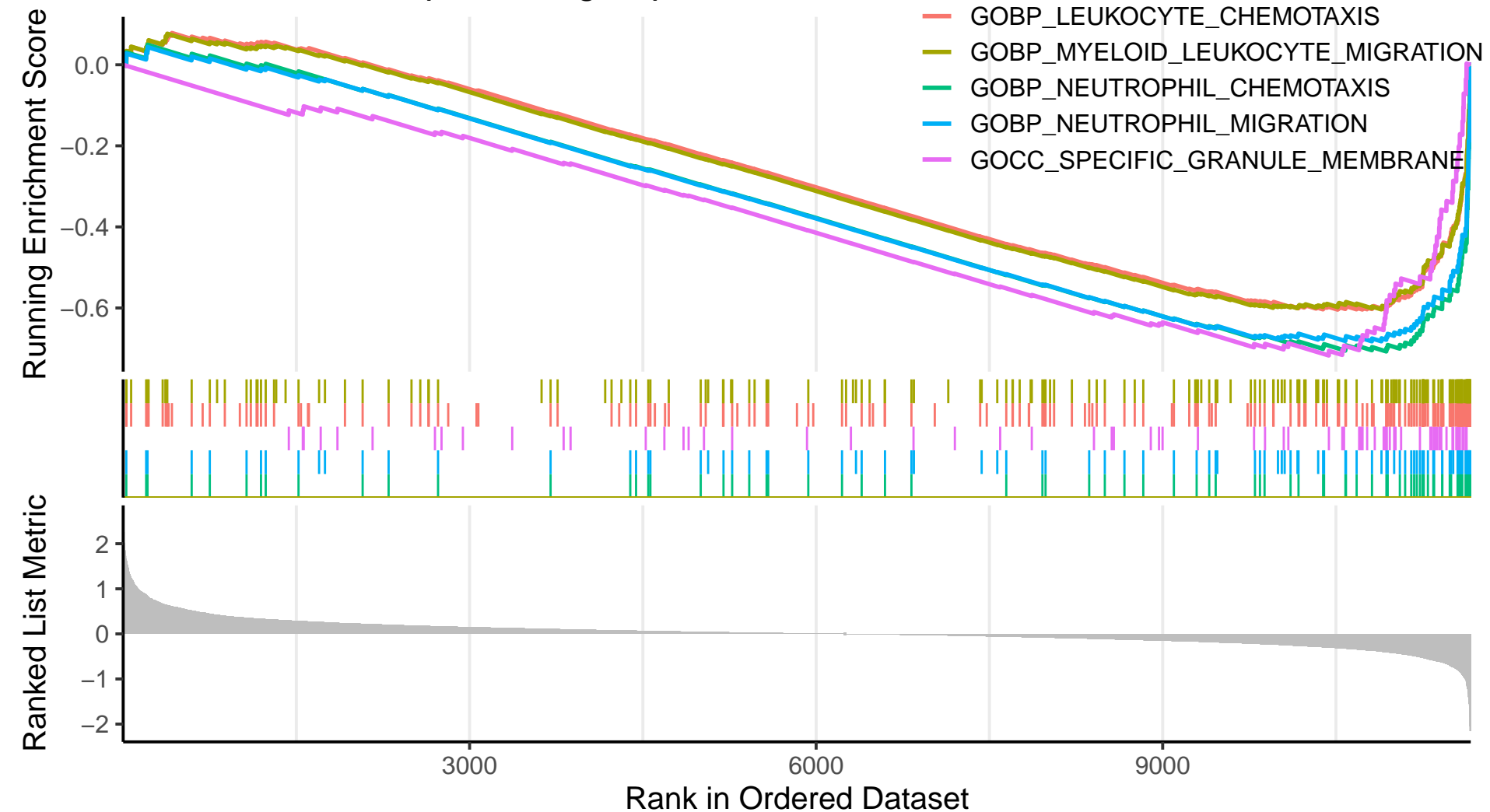

Supplement: S2 File — (ZIP) [file pone.0328906.s002.zip › Supplementary materials2/FGFR3.lowExp.pdf]

# Enriched in high expression group

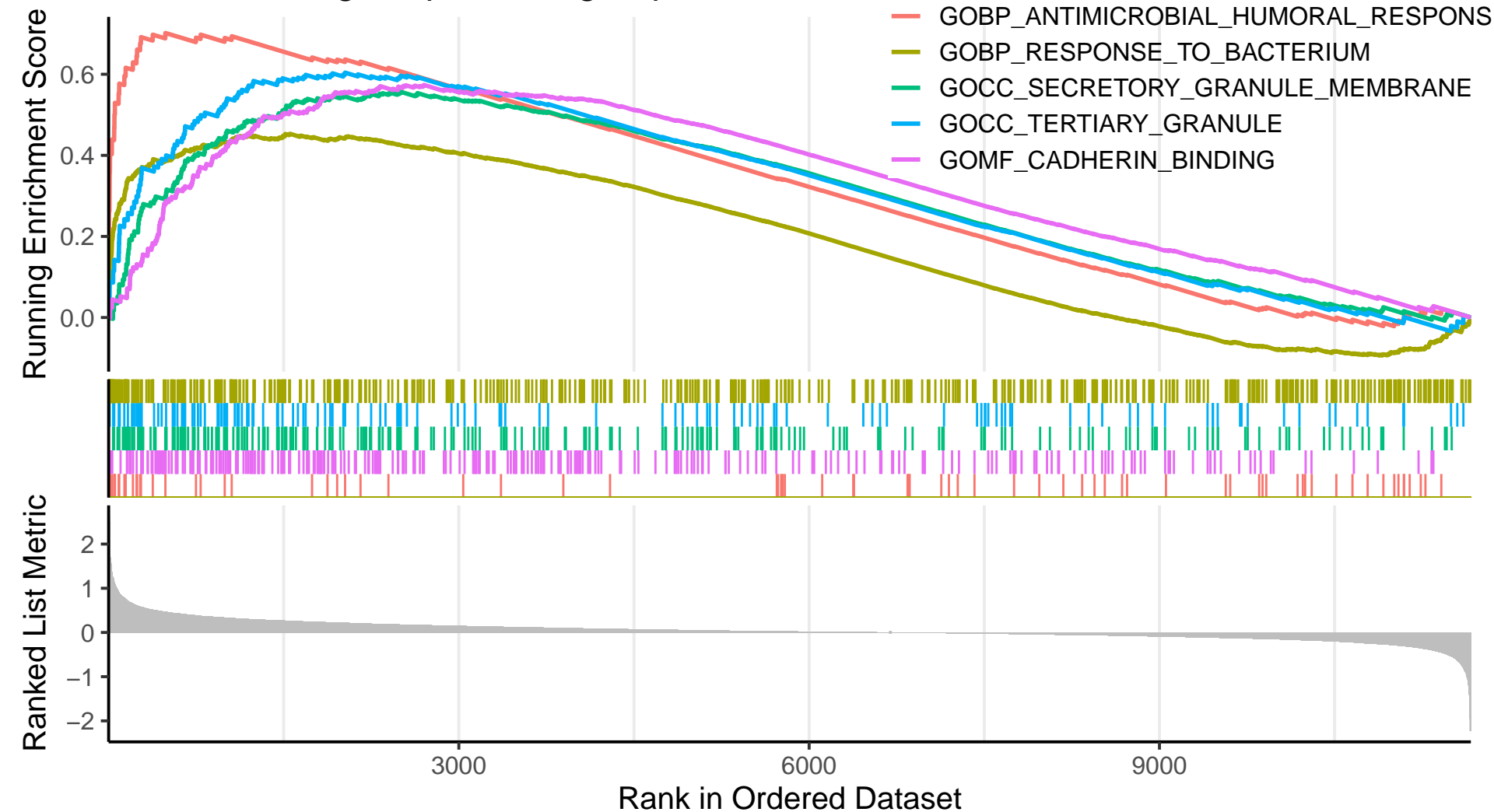

Supplement: S2 File — (ZIP) [file pone.0328906.s002.zip › Supplementary materials2/PI3.highExp.pdf]

# Enriched in low expression group

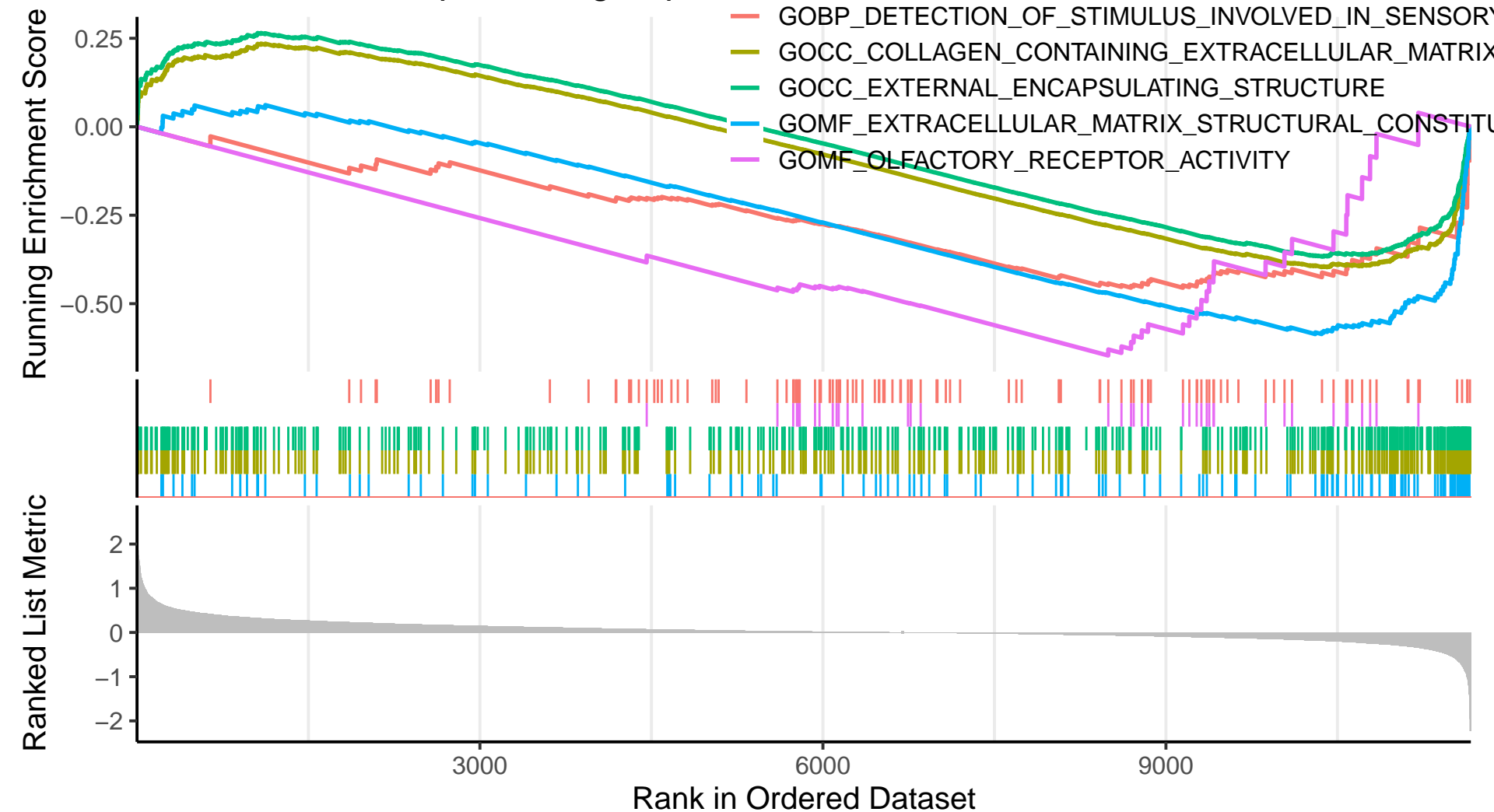

Supplement: S2 File — (ZIP) [file pone.0328906.s002.zip › Supplementary materials2/PI3.lowExp.pdf]

# Enriched in high expression group

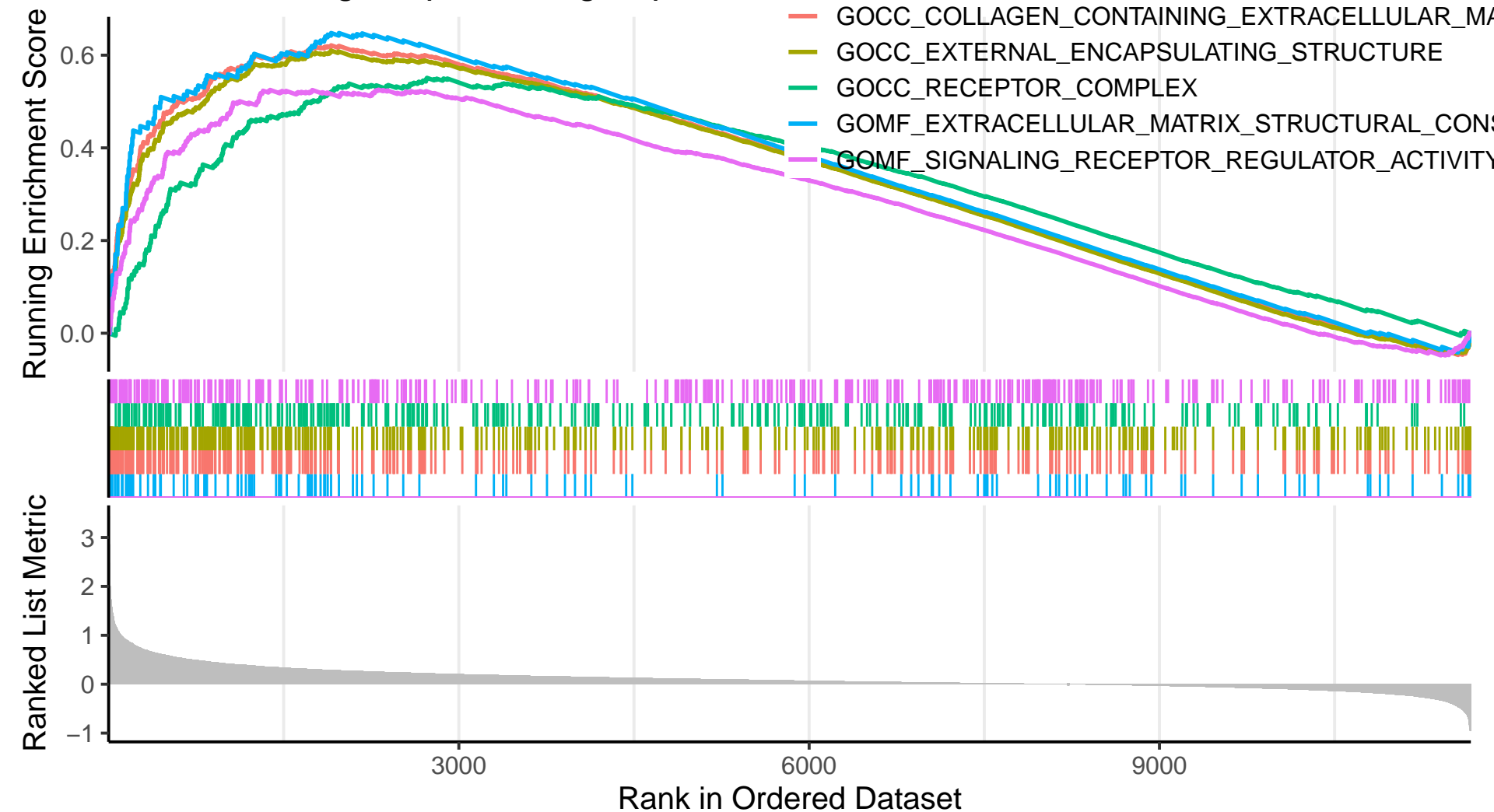

Supplement: S2 File — (ZIP) [file pone.0328906.s002.zip › Supplementary materials2/PLA2G2A.highExp.pdf]

# Enriched in low expression group

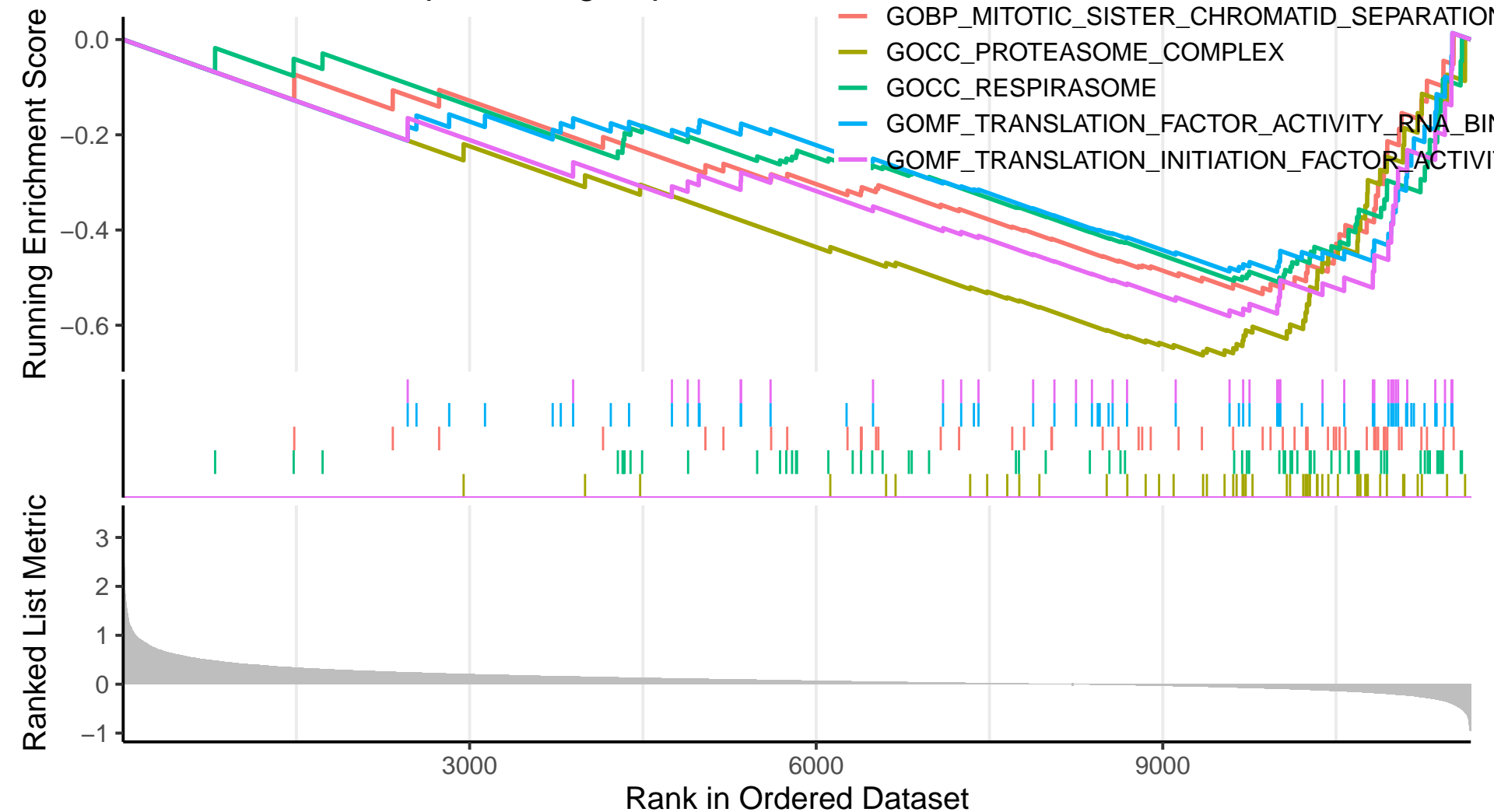

Supplement: S2 File — (ZIP) [file pone.0328906.s002.zip › Supplementary materials2/PLA2G2A.lowExp.pdf]

# Enriched in high expression group

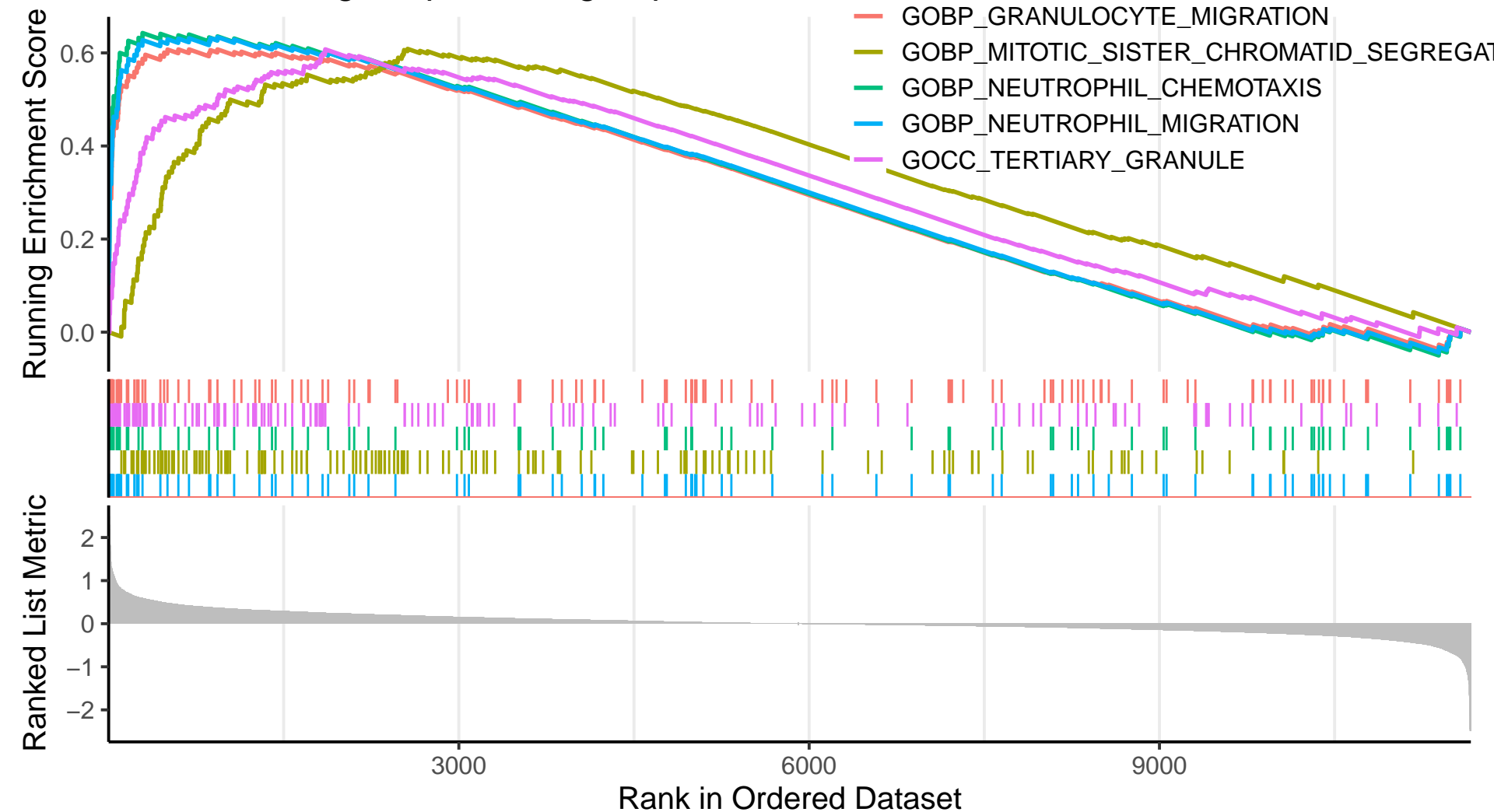

Supplement: S2 File — (ZIP) [file pone.0328906.s002.zip › Supplementary materials2/S100A8.highExp.pdf]

# Enriched in low expression group

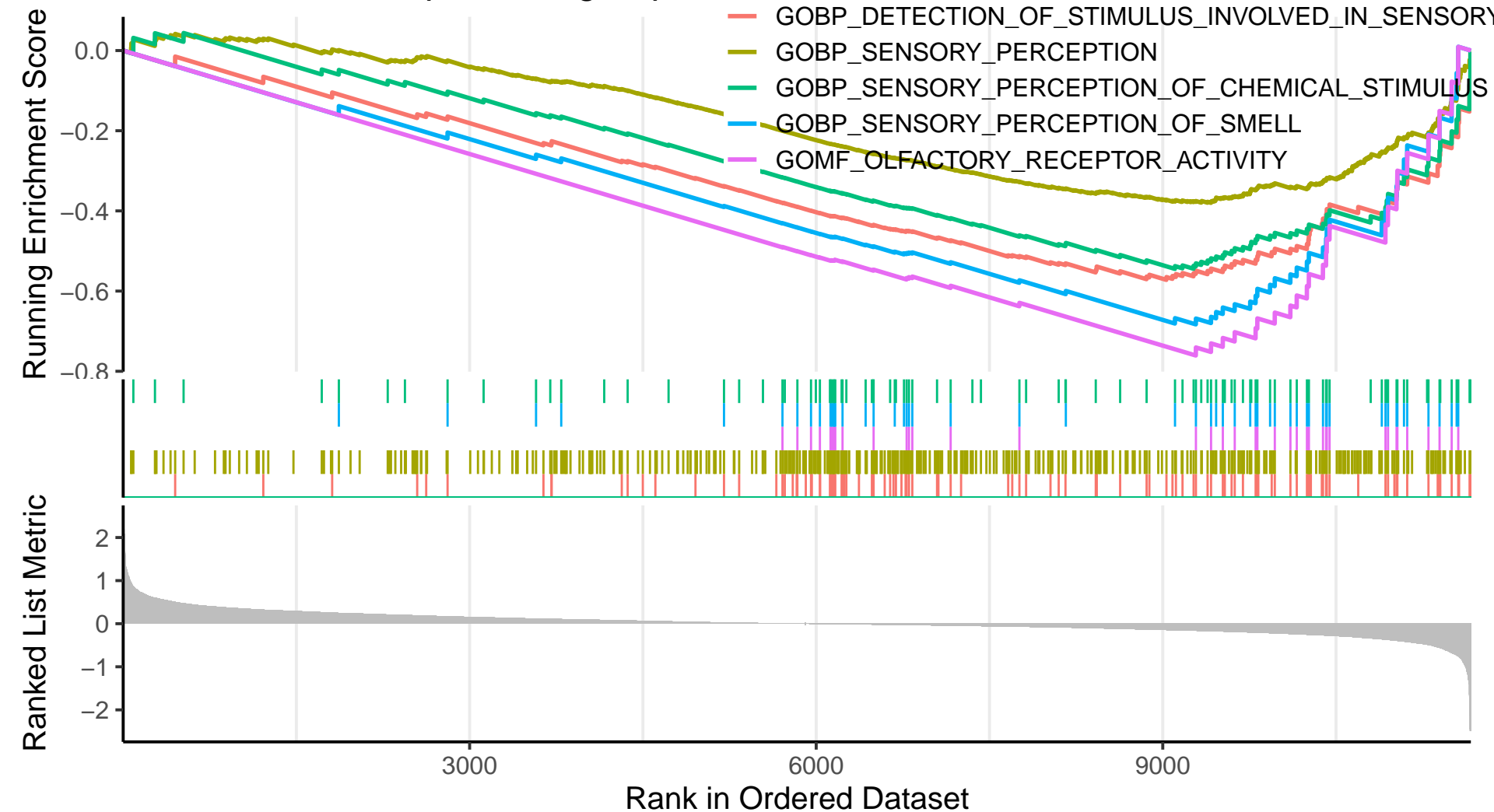

Supplement: S2 File — (ZIP) [file pone.0328906.s002.zip › Supplementary materials2/S100A8.lowExp.pdf]
